# In‐Plate Chemical Synthesis of Isopeptide‐Linked SUMOylated Peptide Fluorescence Polarization Reagents for High‐Throughput Screening of SENP Preferences

**DOI:** 10.1002/cbic.202200601

**Published:** 2022-12-07

**Authors:** Yara Huppelschoten, Rishov Mukhopadhyay, Jens Buchardt, Thomas E. Nielsen, Alfred C. O. Vertegaal, Huib Ovaa, Gerbrand J. van der Heden van Noort

**Affiliations:** ^1^ Dept. Cell and Chemical Biology Leiden University Medical Centre Einthovenweg 2 2333 ZC Leiden The Netherlands; ^2^ Global Research Technologies Novo Nordisk A/S Novo Nordisk Park DK-2760 Måløv Denmark; ^3^ Current address: CMC API Development Novo Nordisk A/S Novo Nordisk Park 2880 Bagsværd Denmark

**Keywords:** assay development, chemical protein synthesis, fluorescence polarisation, proteases, solid phase peptide synthesis

## Abstract

Small ubiquitin‐like modifiers (SUMOs) are conjugated to protein substrates in cells to regulate their function. The attachment of SUMO family members SUMO1‐3 to substrate proteins is reversed by specific isopeptidases called SENPs (sentrin‐specific protease). Whereas SENPs are SUMO‐isoform or linkage type specific, comprehensive analysis is missing. Furthermore, the underlying mechanism of SENP linkage specificity remains unclear. We present a high‐throughput synthesis of 83 isopeptide‐linked SUMO‐based fluorescence polarization reagents to study enzyme preferences. The assay reagents were synthesized via a native chemical ligation‐desulfurization protocol between 11‐mer peptides containing a γ‐thiolysine and a SUMO3 thioester. Subsequently, five recombinantly expressed SENPs were screened using these assay reagents to reveal their deconjugation activity and substrate preferences. In general, we observed that SENP1 is the most active and nonselective SENP while SENP6 and SENP7 show the least activity. Furthermore, SENPs differentially process peptides derived from SUMO1‐3, who form a minimalistic representation of diSUMO chains. To validate our findings, five distinct isopeptide‐linked diSUMO chains were chemically synthesized and proteolysis was monitored using a gel‐based read‐out.

## Graphical abstract

**In‐plate chemical synthesis** of a set of fluorescent SUMO3‐peptide conjugates using a native chemical ligation‐desulfurization strategy is described. These fluorescence polarization reagents were used to screen and reveal the activity and substrate preferences of five SUMO‐proteases (SENPs) in a high‐throughput manner.

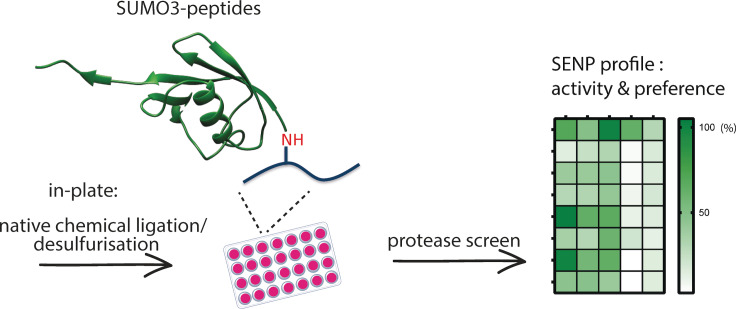

## Introduction

The small ubiquitin‐like modifier (SUMO) protein, similar to ubiquitin (Ub), is a post‐translational modification attached to lysine residues in target proteins. SUMOylation affects thousands of predominantly nuclear proteins in mammalian cells and is, therefore, a key event in many nuclear processes.[^1^, ^2^, ^3^] There are at least four mammalian SUMO‐isoforms, with most common isoforms classified as SUMO1 (with 50 % sequence similarity to SUMO2/3), and SUMO2/3 since mature SUMO2 and SUMO3 differ only by three amino acids and hence are virtually identical.^[4]^ SUMO4^[5]^ and SUMO5^[6]^ have been discovered more recently and are not as ubiquitously expressed as SUMO1, 2/3, and further studies are necessary to shed light on the involvement of these proteins in basic cellular processes. SUMO5 could represent a SUMO pseudogene. Protein SUMOylation results in a diverse and broad range of cellular effects ranging from regulating subcellular protein localization to transcription factor activity, protein stability and cell stress responses.^[7]^

SUMO conjugation is performed by an enzymatic cascade consisting of an E1 activating enzyme (SAE‐2/1), an E2 conjugating enzyme (Ubc9 also known as UBE2I) and a limited number of E3 ligases.^[8]^ The first step involves the ATP‐dependent activation of the SUMO C‐terminal carboxylate resulting in the formation of an E1∼SUMO thioester followed by a trans‐thioesterification reaction with the active site cysteine of Ubc9 to form the E2∼SUMO thioester. If substrates contain a SUMO conjugation motif ΨKXE, where Ψ is a large hydrophobic amino acid and X is any amino acid, Ubc9 is capable of directly SUMOylating the target in the absence of an E3 ligase.^[8]^ Otherwise, an E3 ligase mediates the transfer to the lysine ϵ‐amine of the target protein.^[4]^ SUMO can be attached to a single lysine residue (mono‐SUMOylation) or to multiple residues (multi‐mono‐SUMOylation) on its target protein. SUMO1 is mainly conjugated to proteins as a monomer, however, SUMO2/3 can also form SUMO‐polymers by forming isopeptide‐linkages between the lysine residues of an initial (proximal) SUMO and the C‐terminus of the following (distal) SUMO.^[9]^ Lysine 11 is the preferred residue for the formation of SUMO2/3 chains, since it is situated in a SUMO consensus site.[^9^, ^10^] However, SUMO2/3 chains can also be formed via e. g. lysine5, lysine21, or lysine35.^[10]^

SUMO conjugation is reversible; deconjugation is performed by cysteine proteases called SUMO‐specific isopeptidases. The SUMO‐isopeptidases are classified into three distinct families: the Ulp/SENP (ubiquitin‐like protease/sentrin‐specific protease) family, the DeSI (deSUMOylating isopeptidase) family and USPL1 (ubiquitin‐specific peptidase‐like protein 1). Dysregulation of these proteases has been associated with several diseases and SENPs are consequently interesting targets for drug development.[^11^, ^12^] Mammalian cells express six distinct SENPs; SENP1, SENP2, SENP3, SENP5, SENP6 and SENP7, that all contain a conserved protease domain with a traditional catalytic triad (Cys‐His‐Asp).^[13]^ All SENPs exhibit isopeptidase activity to cleave the isopeptide bond between the glycine residue of SUMO and the lysine side chain of the substrate protein.^[14]^ In addition, they all are structurally organized into a non‐conserved N‐terminal region and a C‐terminal catalytic domain.[^13^, ^15^] The N‐terminal regions of the SENPs contain structural elements responsible for governing the subcellular localization of the proteases and are thought to be also involved in dictating their SUMO‐paralogue and substrate specificities. SENP6 and SENP7 are closely related to their yeast ortholog ULP2, which has a preference for processing of polymers, while all other SENPs are related to the yeast ortholog ULP1.^[16]^ In analogy to the yeast ULPs, mammalian SENPs indeed show preferences for the deconjugation of mono‐SUMOylated proteins or the disassembly of polymeric SUMO chain paralogues.[^17^, ^18^] SENP6 and SENP7 prefer SUMO2/3 chain depolymerization over SUMO2/3 deconjugation as expected.[^19^, ^20^] SENP1 is categorized as the least specific SENP and besides maturing all SUMO precursors also cleaves SUMO1 conjugates next to SUMO2/3 conjugates.[^16^, ^21^] SENP2 has limited ability to discriminate between SUMO paralogues in deconjugation.^[16]^ In addition, SENP3 and SENP5 both prefer SUMO2/3 deconjugation over SUMO1 deconjugation. However, in depth knowledge about intrinsic SENP differences, preferences for SUMO‐paralogues and mechanisms underlying SUMOylated substrate specificity are still enigmatic.

**Figure 1:**
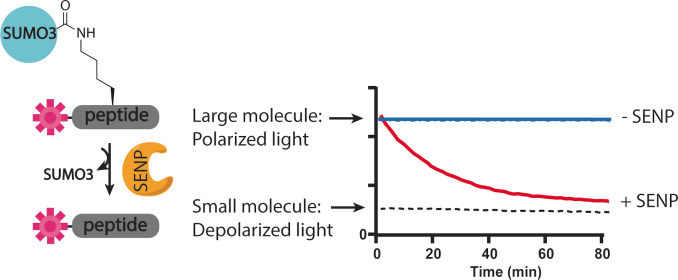
FP assay. When a fluorophore, covalently attached to a small peptide is excited by polarized light, it will mainly emit depolarized light. When it is attached to a larger protein (e. g., SUMO) the emitted light is less depolarized. The activity of the enzyme can be monitored by following the change in fluorescence polarization.

In contrast to the extensive knowledge on deubiquitinating enzymes (DUBs) we are very limited in our understanding of SENPs substrate specificity. Biochemical analysis and crystal structures have been instrumental in the discovery of linkage specificity of several DUBs,[^22^, ^23^, ^24^, ^25^] and significant contributions to profile DUBs have come from the development of assay reagents/probes.^[26]^ For example, in previous work by Geurink et al. DUB specificity was investigated by using isopeptide‐linked fluorescent assay reagents.^[27]^ Fluorescently labeled minimal substrate dimer peptides were enzymatically conjugated to Ub, Nedd8 and SUMO to form an iso‐peptide bond and used as reagent in fluorescence polarization (FP) assays to investigate the specificity of several DUBs and SENPs.^[27]^ A more sophisticated set of FP‐assay reagents, based on peptides derived from different Ub‐linkage sites, was used to determine Ub‐isotype linkage specificity of the ovarian tumor protease (OTU) DUB family.^[22]^ We envisioned that a similar approach using isopeptide‐linked SUMO fluorescence polarization assay reagents, carrying elongated peptide parts would be fitting to shed light on the substrate recognition and associated cleavage specificity of SENPs that might be (co‐)regulated by the substrate's sequence context (Figure 1). Since FP‐assays can be read out in real‐time in multi‐well plate format, we envisioned that a high‐throughput screen containing a large number of assay reagents would be optimal to gain as much information as possible in an efficient manner. For this an in‐plate native chemical ligation‐desulfurization strategy for the synthesis of FP assay reagents in 96‐well format was designed. To have a diverse and relevant set of peptide sequences 96 of the highest SUMOylated proteins found in a proteomics screen by Hendriks et al. were taken and 11‐mer peptides derived from these SUMOylation sites were designed.^[28]^ Native chemical ligation (NCL) to SUMO3‐thioester followed by desulfurization in 96‐well format resulted in the successful generation of 83 assay reagents, whereas 13 SUMO3‐peptide conjugates could not be isolated in sufficient amounts to quantify. Subsequently the 83 FP‐reagents were used in the high‐throughput screen against the catalytic domains of 5 of the 6 human SENPs (SENP1, SENP2, SENP5, SENP6, and SENP7). To validate a small portion of the FP‐screening results we synthesized six different native diSUMO chains to explore whether expanding the substrate context from SUMO3‐SUMO peptide conjugate to SUMO3‐SUMO protein conjugate would impact proteolytic preferences. We applied an NCL approach using a γ‐thiolysine‐equipped SUMO mutant with the SUMO3‐thioester followed by radical desulfurization, all under native buffered conditions, and finally performed SENP1 and SENP6 mediated proteolysis experiments on the purified SUMO dimers.

## Results and Discussion

### Synthesis design

We started with the design and synthetic strategy of the probes. The substrate peptides must be small to obtain an optimal FP‐effect but still contain enough peptide length to provide sufficient context for the SENPs to be able to discriminate and convey their preferences. Therefore, substrate peptides containing 11 amino acids were designed, resulting in five substrate‐derived amino acids on the C‐terminal‐ and on the N‐terminal side of the iso‐peptide linked SUMOylated lysine residue. The small size of the 11‐mer peptides (∼1.2 kDa) would lead to an optimal FP range compared to the larger SUMO3 protein (∼10 kDa). To make these peptides suitable for FP‐measurements they were functionalized on the N‐terminus with a 5‐carboxytetramethylrhodamine (TAMRA) fluorophore via a PEG2 spacer to prevent blockage of SENP active sites through hydrophobic interactions with the fluorophore.

To selectively SUMOylate the target lysine, we envisioned that a one‐pot NCL and desulfurization approach between a γ‐thiolysine containing peptide and a SUMO3 thioester (peptide 1) would be the most effective approach.^[29]^ Concentration, pH and thiol catalyst are essential reaction parameters during NCL and challenging to control simultaneously in 96‐well plate format, thus a robust and optimized NCL protocol was required.^[30]^ To add an additional challenge to this synthesis we aimed to perform the reaction under native conditions as Bouchenna et al. observed that the native cysteine residue in SUMO3 is important for its folding and recognition by SENPs.^[31]^ By performing the desulfurization under native conditions (phosphate buffer) this cysteine remains unaffected in contrast to desulfurization under denaturing conditions.

### Synthesis of fluorescent polarization assay reagents

95 out of the envisioned 96 peptides (Table S1) were efficiently synthesized in parallel on solid support, followed by reversed‐phase purification and lyophilization to yield pink solids in a 96‐well format (Table S2). To obtain a sufficient amount of reagent for screening of the five SENPS the aim was to perform the NCL at 2.5 mg SUMO3 per well, requiring a minimal total amount of 240 mg SUMO3 thioester. Large scale synthesis of SUMO3 was performed on hydrazine resin followed by acid promoted cleavage and thioester formation (Figure S1).^[32]^ Next, we set out to investigate several conditions for the NCL and desulfurization in the envisioned 96‐well format. We used peptide A4, a peptide derived from RANGAP1, as test peptide. After extensive testing 2‐mercaptoethanesulfonic acid (MESNa) proved to be the most suitable thioester and catalyst during NCL with γ‐thiolysine. Due to the higher stability of the MESNa thioester compared to 4‐mercaptophenylacetic acid (MPAA) or trifluoroethanethiol (TFET)^[33]^ thioesters, less hydrolysis was observed and higher yields obtained. In addition, the aliphatic MESNa is compatible with an one‐pot NCL and desulfurization strategy, in contrast to aromatic MPAA, in which it can be used as hydrogen donor in the radical desulfurization reaction.^[34]^

**Figure 2:**
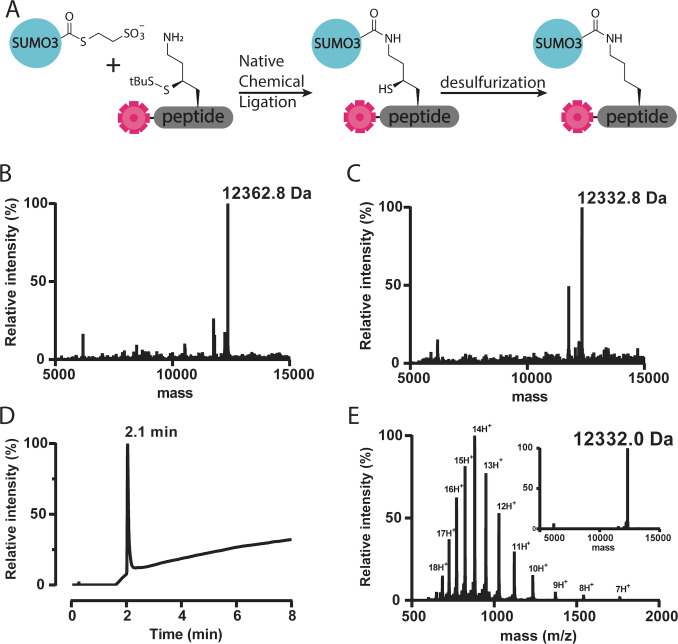
A. Reaction scheme for the NCL and desulfurization in 96‐well format. B–E. representative example of LC–MS analysis of NCL of reagent H6. B. Deconvoluted mass of crude NCL mixture H6. Calculated: 12361.8, observed: 12362.8. C. Deconvoluted mass of desulfurized H6. Calculated: 12331.0, observed: 12332.8. D. Total ion chromatogram of reagent H6, Rt 2.1 min. E. ESI spectrum of purified probe H6 and deconvoluted mass of purified H6. Calculated: 12331.0, observed: 12332.0.

With all 95 TAMRA and γ‐thiolysine equipped peptides and SUMO3‐thioester in hand we commenced the reaction in the 96‐well plate. Prior to the ligation the peptides were first dissolved and incubated for 30 minutes in phosphate buffer containing 1 M tris(2‐carboxyethyl)phosphine (TCEP) to reduce the StBu protecting group of the γ‐thiolysine, making it susceptible for NCL. Next, the SUMO3‐thioester (peptide 1) was added in phosphate buffer to obtain a final concentration of 1 mM SUMO‐thioester and 100 mM TCEP. After 16 hours of incubation at 37 °C the reactions had reached completion as shown by LC–MS analysis (Figure 2). For the subsequent one‐pot desulfurization, the NCL mixture was diluted with a mixture of TCEP and radical initiator VA‐044, using the already present MESNa from the ligation as hydrogen donor. After 16 hours at 40 °C LC–MS analysis showed completion and importantly only single desulfurization for 88 out of 95 reagents (Figure 2 and Table S3). In addition six reactions were found to show double desulfurization, which we attribute to additional desulfurisation of the cysteine residue in the peptide sequence, while one reaction did not show appreciable desulfurization at all. After RP‐HPLC purification 83 iso‐peptide linked SUMO3 FP‐peptide conjugates could be obtained with an average overall yield of 3.3 % (Figure 2 and Table S3). To verify the observed desulfurisation took place on the desired thiolysine position while leaving the native cysteine of SUMO3 unaffected, we incubated a mono desulfurised and double disulfurised SUMO3‐peptide with SENP1 to cleave the isopeptide bond between SUMO3 and the peptide. LC–MS analysis revealed that indeed desulfurisation occurred on the peptide and the SUMO3 protein retained its cysteine residue (Figure S2 and S3).

### High‐throughput screening of 83 FP assay reagents

Next, the FP reagents were tested in deconjugation assays by treatment with five different SENPs (SENP1, SENP2, SENP5, SENP6, and SENP7). Expression efforts to recombinantly obtain SENP3 failed, therefore this isopeptidase unfortunately could not be taken along in the screen. First, the proteases were incubated at six different concentrations with the positive control and minimal substrate TAMRA‐K^SUMO3^G (Figure S4) to determine the optimal concentration for linear reaction progress in a convenient time window (60–90 minutes) (Table S4). Subsequently, a time course experiment of all 83 probes with one single SENP concentration was performed (three representative examples are shown in Figure S5–7). To be able to compare data, all probe signals were normalized against the control substrate TAMRA‐K^SUMO3^G signal to obtain a percentage of processing (Table S5).

In general, we observed that SENP1 is the most active and non‐selective SENP, with 64 probes processed above 50 % compared to the positive control (TAMRA‐K^SUMO3^G). SENP6 and SENP7 showed the least activity towards the probes, while good processing of the positive control was observed. Both SENP6 and SENP7 are known to prefer depolymerization of SUMO polymers, hence this observation is not unexpected.[^20^, ^35^] Although, SENP6 is considered to have no C‐terminal hydrolase activity^[16]^ our data does show that SENP6 is able to cleave small peptides from the C‐terminus of SUMO3.

Interestingly, probe F7 was one of the few not processed by SENP1 (only 2 %), that did show relatively good processing by SENP6 (30 %) and SENP7 (25 %). The peptide of probe F7 is derived from the chromobox 3 (CBX3) protein, that binds DNA and is a component of heterochromatin. It also is an interaction partner of CBX5, which is known to interact with SENP7. In previous work, GST‐SENP7 was able to pull‐down CBX3 (HP1γ) from nuclear extract, confirming a possible interaction between SENP7 and CBX3.^[36]^ These observations warrant future detailed studies to verify whether SENP7 and SENP6 indeed are involved in deSUMOylation of CBX3.

Another general observation that can be made from the FP‐screen is that all probes that are poorly processed by SENP1 contain a high amount of charged amino acids. Four out of the five peptides that score a conversion of less than 40 % contain six or more charged amino acids in the sequence. This might indicate that the active site of SENP1 is not compatible with charged peptides due to electrostatic repulsion. Furthermore, all probes that are processed well by SENP6 are typically processed even better by SENP5, with the exception of probe F7. Again, maybe indicating a repulsion of this peptide in the active site of SENP5, but not SENP6.

Five out of the top ten best processed probes by SENP6 in our FP‐screen, namely SUMO3 K41 (C9), SP100 K297 (F3), PML K160 (B8), PARP1 K203 (G4), and PIAS1 K137 (H3) (Table S5), were recently also identified in a proteomics screen performed by Liebelt et al. that focused on polySUMOylated proteins which depend on SENP6 for deconjugation.^[37]^ It sparked our interest to find that hits from the FP assay are processed by SENP6 in vivo. Furthermore, out of the four probes B7 (K486), D5 (K512), F10 (K467) and G4 (K203) derived from PARP1 only G4 was processed very well (40 %, compared to 5 %, 10 % and 20 % for the other probes, respectively) by SENP6 (Table S5). This might be an indication that SUMO3ylation of K203 on PARP1 occurs and that this could be a preferred target for deconjugation by SENP6. Further validation, however, is required to determine if deSUMOylation of lysine203 of PARP1 in vivo is indeed occurring and (solely) dependent on SENP6 however, as the other SENPs are also able to process the FP‐probe G4.

It has been reported that SENP6 is responsible for the proteolysis of SUMO polymers conjugated to the target protein promyelocytic leukemia protein (PML).[^16^, ^38^] Three of our probes were derived from PML; lysine490, lysine160, and lysine380. Interestingly, SENP6 efficiently processed the probe derived from lysine160 while lysine490 and lysine380 were processed significantly less (Table S5).

Another interesting observation made is the probe derived from spartan protein (SPRTN) (E4) that is mainly processed by SENP5. The other four SENPs do not process this probe efficiently, raising the question whether deconjugation of SUMO3 from SPRTN is specifically carried out by SENP5. Further investigation, however, is needed to validate these results.

**Figure 3:**
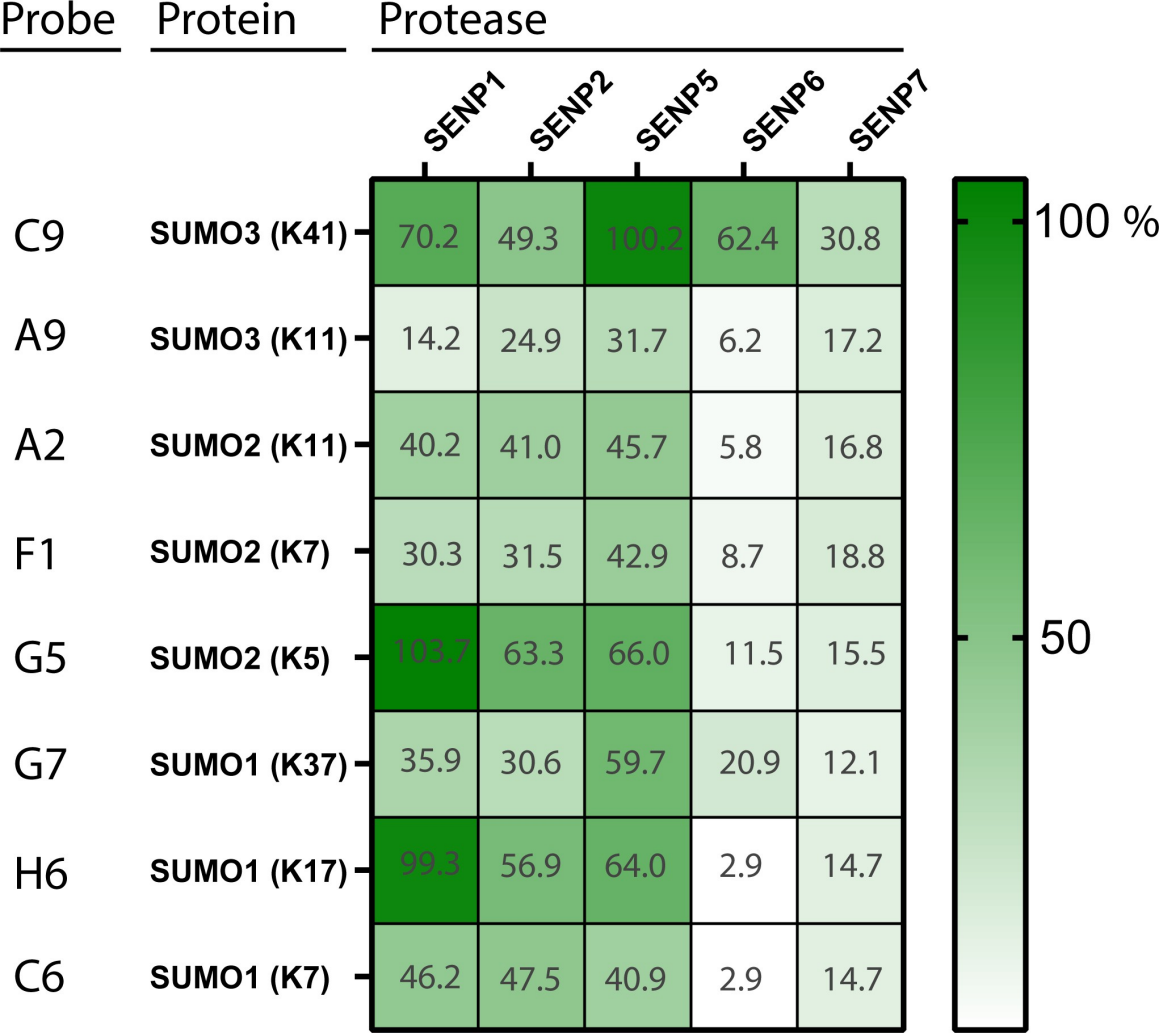
Results of FP assay of SUMO‐derived peptides.

Peptides derived from SUMO1, SUMO2, and SUMO3 form a minimalistic representation of a diSUMO chain. Interestingly, they are processed differentially by the various SENPs. The SUMO2 K11 derived peptide (A2) is processed mediocre by SENP1, 2 and 5, but rather poorly by SENP6 and SENP7. This is an interesting observation as SENP6 and SENP7 are known to prefer SUMO2/3 chain depolymerization.^[19]^ Perhaps the 11‐mer peptide does not provide enough sequence context to mimic a SUMO polymer, or SUMO polymer deconjugation by these SENPs relies more heavily on additional binding motifs in SUMO located further away from the isopeptide‐linkage region. Another observation is the difference in processing of the SUMO3 K11 derived peptide (A9) and the SUMO2 K11 derived peptide (A2) even though only different by two amino acids (Figure 3). SENP1, 2 and 5 remarkably lose most of their ability to proteolyze A9 compared to A2. Furthermore, probe C9 SUMO3 K41 is processed very efficiently by both SENP1, SENP5 and SENP6 however a second probe derived from SUMO3 K11 (A9) was processed less efficiently by these SENPs, indicating that perhaps not only distinct SUMO‐isoforms but also different SUMO‐linkage types can be preferentially recognized by some SENPs.

### Isopeptide‐linked diSUMO proteolysis

The initial FP‐assay was performed using a fixed enzyme and fixed substrate concentration. To validate these results and simultaneously establish that proteolysis of the FP‐probes is enzyme concentration dependent, a second FP assay was performed with fixed substrate concentrations against increasing SENP concentrations (Figure S8–S12). We selected the SUMO derived peptides (see Figure 3) and indeed this assay reflected the outcome of the values obtained in the single concentration FP assay and showed a faster or more pronounced FP‐response upon increasing SENP concentration.

To investigate whether SENP specificity mainly arises from the sequence surrounding the iso‐peptide bond linkage or is dependent on the entire proximal SUMO we wanted to establish the SENP mediated proteolysis profiles of different diSUMO linkages with gel‐based assays. K11 linked SUMO3/SUMO2 dimers have been chemically prepared previously via a crypto‐thioester native chemical ligation approach or an elaborate auxiliary mediated ligation strategy.[^39^, ^40^] Other lysine linkages have not been prepared chemically and we deemed the one‐pot non‐denatured thiolysine mediated NCL‐desulfurization approach, as was used for the FP‐probes in the 96 well format, suitable.

**Scheme 1:**
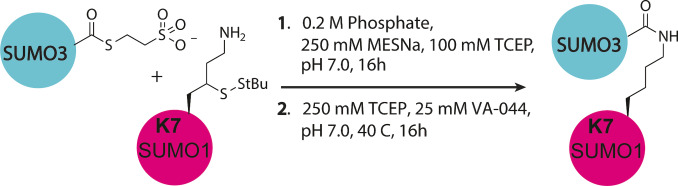
Schematic representation of the synthesis of SUMO3 – SUMO1 dimer linked via K7 in SUMO1.

**Figure 4:**
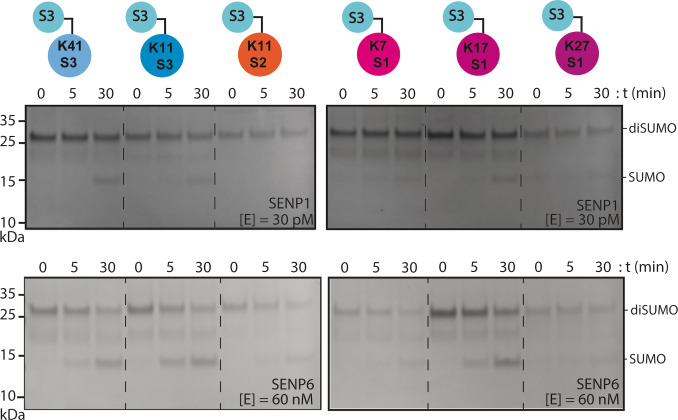
Specificity analysis of SENP1 and SENP6 cleavage of S3‐S3 K41, S3‐S3 K11, S3‐S2 K11, S3‐S1 K7, S3‐S1 K17, and S3‐S1 K37. Coomassie stained gels. Top gels: SENP1 was used at 30 pM and after 0, 5, and 30 minutes 2 μg of the protein mixture was taken for analysis. Bottom gels: SENP6 was used at 60 nM and after 0, 5, and 30 minutes 2 μg of the protein mixture was taken for analysis. S1=SUMO1, S2=SUMO2, S3=SUMO3.

We hence synthesized six different diSUMO linkages (Figure S19–25) using γ‐thiolysine SUMO mutants (Figure S13–S18) and SUMO3 thioester (Scheme 1, Table S6–S7) and successfully prepared SUMO3 linked to SUMO3 K41, SUMO3 K11, SUMO2 K11, SUMO1 K7, SUMO1 K17 and SUMO1 K37. Next, linkage specific cleavage of the different diSUMOs by SENP1 and SENP6 was followed over time in a gel‐based assay. The dimers were incubated with the SENP at the indicated concentrations and samples were taken at different time points. After 30 minutes SENP1 partially cleaved SUMO3 K41‐ and SUMO1 K17‐dimers, but also S1 K7‐, S1 K37‐ and S3 K11‐dimers were processed albeit at an apparent lower rate (Figure 4). Although this gel‐based assay is not quantitative, the apparent preference for SUMO3‐K41 and SUMO1‐K17 over all other linkages reflects the outcome of the initial FP‐assay (Figure 3). In addition, when a higher SENP1 concentration is used non‐specific cleavage is observed as was reported by Mevissen et al.^[22]^

Next, SENP6 was tested on the diSUMO chains to verify that FP probe C9 (SUMO3 K41) was efficiently processed by SENP6 in contrast to all other diSUMO based FP probes. The S3‐S3 K41 dimer is indeed also processed well by SENP6 in the gel‐based assay, however S3‐S3 K11 and S3‐S1 K17 were observed to be cleaved at least equally well (Figure 4). It is well known that SENP6 prefers the cleavage of SUMO2/3 polymer, hence processing of the S3‐S3 K11 dimer was not unexpected. However, processing of S3‐S1 K17 is unexpected since SENP6 is reported to not have a SUMO1 preference.^[38]^ The unexpected processing of S3‐S1 K17 could be a non‐specific observation since only the catalytic domain of SENP6 is used. Furthermore, whether extending the FP‐peptides to full SUMO dimers is sufficient to generate biological relevant cleavage profiles needs further investigation as SENP6’s function is mainly attributed to SUMO chain proteolysis. However, it is interesting that in these in vitro assays on diSUMO chains as well as the FP‐SUMO assay SENP6 shows activity and selectivity towards a subset of the used reagents.

## Conclusion

To conclude, we successfully developed a high throughput one‐pot NCL and desulfurization protocol for the synthesis of isopeptide‐linked SUMO3 assay reagents in a multi‐well plate format. The NCL was performed under native conditions to protect the native cysteine residue from the desulfurization reaction conditions. Eighty three out of the 96 probes were successfully synthesized and purified followed by a screen against the C‐terminal catalytic domains of five SENPs. Using this simple in vitro approach, a large dataset containing SENPs preferences was generated. Expansion of the current suite of SUMO3‐FP probes with FP‐probes based on SUMO1 could further establish specific profiles of and insights in SENP preferences. Further in vitro and in vivo validation of the current data is necessary to confirm SENP preferences, however, some interesting observations were made that warrant future, in‐detail investigations. The data collected provide a global perspective on the relative activities of the different SENPs. Conduction of similar FP assays with full length SENPs could provide additional information about the influence of the N‐terminal SENP domains with respect to their specificities and help in unraveling the molecular details and cell biology of these proteases.

## Experimental Section

**Synthesis in 96‐well plate format**: The synthesis was performed using an automated peptide synthesizer (Intavis, Multipep CF) using standard 9‐fluorenylmethoxycarbonyl (Fmoc) based SPPS. Fmoc deprotection was achieved with 2×10 min. treatment of 20 vol.% piperidine, 0.1 % Oxyma Pure® in DMF. Peptide couplings were performed using DIC/Oxyma Pure®. Amino acid/Oxyma Pure® solutions (0.3 M/0.3 M in DMF) were added to the resin at 4‐fold excess together with equal equivalents of DIC (1.5 M in DMF). Triple couplings were performed with a coupling time of 1 hour. After the final Fmoc deprotection the resin was washed with DMF and DCM.

**Global deprotection from the resin and side chain deprotection**: Polypeptide sequences containing a cysteine residue were detached from the resin and deprotected by treatment with Reagent K (TFA/phenol / H_2_O/thioanisole/EDT, 82.5 : 5 : 5 : 5 : 2.5 v/v/v/v/v) for 2–3 hours followed by precipitation in ice cold diethylether and collection by centrifugation. The pellet was resuspended in diethylether before being collected by centrifugation again. The pellet was dissolved in H_2_O/CH_3_CN/AcOH, 65 : 25 : 10, v/v/v and lyophilized before purification.

**Purification of peptides**: Preparative purification was performed on a Gilson HPLC system using a Phenomenex, Luna 100 Å, C8(2), 10 μm, 30 mm×250 mm column. Elution was performed using 2 mobile phases: A=0.1 % TFA in MilliQ water and B=0.1 % TFA in acetonitrile using a linear gradient. Fractions were collected using a Gilson fraction collector and relevant fractions were assessed by analytical LC–MS. Fractions containing the pure peptide were pooled and lyophilized.

**Assembly of the SUMO probes**: Peptides were dissolved in 1 M TCEP (10 % of the final volume) and incubated at RT for 30 minutes to remove the StBu protection group from the γ‐thiolysine prior to the NCL. Thereafter, followed by the addition of SUMO3 thioester (90 % of the final volume) in 0.2 M Na_2_HPO_4_, 250 mM MESNa and 0.15 M NaCl pH 7.95 buffer, leading to a final concentration of 1 mM SUMO3. The NCL was shaken at 37 °C for 16 hours before LC–MS analysis was taken of all reactions. Followed by the addition of an equal amount of 0.2 M Na_2_HPO_4_, 500 mM TCEP and 50 mM VA‐044 in MilliQ, resulting in a final buffer concentration of 0.2 M Na_2_HPO_4_, 250 mM TCEP, 125 mM MESNa and 25 mM VA‐044 in MilliQ. The reaction was shaken at 350 rpm at 40 °C for 16 hours before LC–MS analysis was taken of all reactions. The volumes of each reaction were adjusted to 1 mL per well before purification by semi‐preparative HPLC using a Phenomenex, Aeris™ widepore 200 Å, C4, 3.6 μm, 4.6×150 mm column followed by lyophilization afforded 83 peptides as pink solids with yields ranging from 0.2 % to 6.6 % with an average yield of 3.3 %.

**Fluorescence polarization SENP assay**: Fluorescent polarization (FP) assays were performed in TRIS buffer (50 mM Tris⋅HCl, pH 7.5, 5 mM DTT, 100 mM NaCl, 1 mg/mL 3‐{dimethyl[3‐(3α,7α,12α‐trihydroxy‐5β‐cholan‐24‐amido)propyl]azaniumyl}propane‐1‐sulfonate (CHAPS) and 0.5 mg/mL bovine gamma globulin (BGG)). All probes (150 nL of 40 μM in DMSO, final concentration 400 nM) were dispensed into ‘‘non‐binding surface flat bottom low flange’’ black 384‐well plates (Corning) plates using an ECHO 550 Liquid Handler (Labcyte Inc.) acoustic dispenser. Buffer was predispensed (10 μL /well) and the reaction was started by the addition of enzyme (SENP1, SENP2, SENP5, SENP6, and SENP7) (5 μL /well, final concentration in Table S6). The plate was centrifuged (1 min at 1,500 rpm) prior to the measurement. FP of the TAMRA fluorophore was measured every 81 seconds for 90 minutes on a Pherastar plate reader (BMG LABTECH GmbH, Germany) with 540‐590‐590 FP module (λ_ex_=540 nm with detection of polarization at λ_em_=590 nm). The obtained data was analyzed by GraphPad prism (version 9.0.1).

Additional data that support the findings of this study are available in the Supporting Information material of this article including peptide sequences, analytical data of SUMO3‐thioester and a selection of SUMO3‐FP peptides, FP‐assays and analytical data of synthesised diSUMOs.

## Conflict of interest

The authors declare no conflict of interest.

1

## Supporting information

As a service to our authors and readers, this journal provides supporting information supplied by the authors. Such materials are peer reviewed and may be re‐organized for online delivery, but are not copy‐edited or typeset. Technical support issues arising from supporting information (other than missing files) should be addressed to the authors.

Supporting Information

## Data Availability

The data that support the findings of this study are available in the supplementary material of this article.

## References

[1] R. Geiss-Friedlander , F. Melchior , Nat. Rev. Mol. Cell Biol. 2007, 8, 947–956. 18000527 10.1038/nrm2293

[2] A. B. Celen , U. Sahin , FEBS J. 2020, 287, 3110–3140. 32255256 10.1111/febs.15319

[3] A. C. O. Vertegaal , Nat. Rev. Mol. Cell Biol. 2022, 23, 715–731. 35750927 10.1038/s41580-022-00500-y

[4] S. Müller , C. Hoege , G. Pyrowolakis , S. Jentsch , Nat. Rev. Mol. Cell Biol. 2001, 2, 202–210. 11265250 10.1038/35056591

[5] D. Guo , M. Li , Y. Zhang , P. Yang , S. Eckenrode , D. Hopkins , W. Zheng , S. Purohit , R. H. Podolsky , A. Muir , J. Wang , Z. Dong , T. Brusko , M. Atkinson , P. Pozzilli , A. Zeidler , L. J. Raffel , C. O. Jacob , Y. Park , M. Serrano-Rios , M. T. Martinez Larrad , Z. Zhang , H. J. Garchon , J. F. Bach , J. I. Rotter , J. X. She , C. Y. Wang , Nat. Genet. 2004, 36, 837–841. 15247916 10.1038/ng1391

[6] Y. C. Liang , C. C. Lee , Y. L. Yao , C. C. Lai , M. L. Schmitz , W. M. Yang , Sci. Rep. 2016, 6, 1–15. 27211601 10.1038/srep26509 PMC4876461

[7] A. Flotho , F. Melchior , Annu. Rev. Biochem. 2013, 82, 357–385. 23746258 10.1146/annurev-biochem-061909-093311

[8] R. T. Hay , Mol. Cell 2005, 18, 1–12. 15808504 10.1016/j.molcel.2005.03.012

[9] N. S. Jansen , A. C. O. Vertegaal , Trends Biochem. Sci. 2021, 46, 113–123. 33008689 10.1016/j.tibs.2020.09.002

[10] A. Gärtner , K. Wagner , S. Hölper , K. Kunz , M. S. Rodriguez , S. Müller , EMBO Rep. 2018, 19, e46117. 30201799 10.15252/embr.201846117 PMC6216285

[11] Y. Jia , L. A. Claessens , A. C. O. Vertegaal , H. Ovaa , ACS Chem. Biol. 2019, 14, 2389–2395. 31361113 10.1021/acschembio.9b00402 PMC6862319

[12] T. Bawa-Khalfe , E. T. H. Yeh , Genes Cancer 2010, 1, 748–752. 21152235 10.1177/1947601910382555 PMC2998238

[13] C. M. Hickey , N. R. Wilson , M. Hochstrasser , Nat. Rev. Mol. Cell Biol. 2012, 13, 755–766. 23175280 10.1038/nrm3478 PMC3668692

[14] E. T. H. Yeh , L. Gong , T. Kamitani , Gene 2000, 248, 1–14. 10806345 10.1016/s0378-1119(00)00139-6

[15] M. Drag , G. S. Salvesen , IUBMB Life 2008, 60, 734–742. 18666185 10.1002/iub.113

[16] R. T. Hay , Trends Cell Biol. 2007, 17, 370–376. 17768054 10.1016/j.tcb.2007.08.002

[17] A. V. Mendes , C. P. Grou , J. E. Azevedo , M. P. Pinto , Biochim. Biophys. Acta 2016, 1863, 139–147. 26522917 10.1016/j.bbamcr.2015.10.020

[18] K. Kunz , T. Piller , S. Müller , J. Cell Sci. 2018, 131, jcs211904. 29559551 10.1242/jcs.211904

[19] K. O. Alegre , D. Reverter , J. Biol. Chem. 2011, 286, 36142–36151. 21878624 10.1074/jbc.M111.268847 PMC3195590

[20] C. D. Lima , D. Reverter , J. Biol. Chem. 2008, 283, 32045–32055. 18799455 10.1074/jbc.M805655200 PMC2581585

[21] L. N. Shen , C. Dong , H. Liu , J. H. Naismith , R. T. Hay , Biochem. J. 2006, 397, 279–288. 16553580 10.1042/BJ20052030 PMC1513277

[22] T. E. T. Mevissen , M. K. Hospenthal , P. P. Geurink , P. R. Elliott , M. Akutsu , N. Arnaudo , R. Ekkebus , Y. Kulathu , T. Wauer , F. El Oualid , S. M. V. Freund , H. Ovaa , D. Komander , Cell 2013, 154, 169–184. 23827681 10.1016/j.cell.2013.05.046 PMC3705208

[23] A. Bremm , S. M. V. Freund , D. Komander , Nat. Struct. Mol. Biol. 2010, 17, 939–947. 20622874 10.1038/nsmb.1873 PMC2917782

[24] K. Keusekotten , P. R. Elliott , L. Glockner , B. K. Fiil , R. B. Damgaard , Y. Kulathu , T. Wauer , M. K. Hospenthal , M. Gyrd-Hansen , D. Krappmann , K. Hofmann , D. Komander , Cell 2013, 153, 1312–1326. 23746843 10.1016/j.cell.2013.05.014 PMC3690481

[25] P. Paudel , Q. Zhang , C. Leung , H. C. Greenberg , Y. Guo , Y. H. Chern , A. Dong , Y. Li , M. Vedadi , Z. Zhuang , Y. Tong , Proc. Natl. Acad. Sci. USA 2019, 116, 7288–7297. 30914461 10.1073/pnas.1815027116 PMC6462090

[26] Y. Huppelschoten , G. J. Van der Heden van Noort , Semin. Cell Dev. Biol. 2022, 132, 74-85. 34961664 10.1016/j.semcdb.2021.11.025

[27] P. P. Geurink , F. El Oualid , A. Jonker , D. S. Hameed , H. Ovaa , ChemBioChem 2012, 13, 293–297. 22213387 10.1002/cbic.201100706 PMC3488293

[28] I. A. Hendriks , A. C. O. Vertegaal , Nat. Rev. Mol. Cell Biol. 2016, 17, 581–595. 27435506 10.1038/nrm.2016.81

[29] G. J. Van der Heden van Noort , R. Kooij , P. R. Elliott , D. Komander , H. Ovaa , Org. Lett. 2017, 19, 6490–6493. 29172548 10.1021/acs.orglett.7b03085 PMC5735377

[30] M. Paolini-Bertrand , F. Cerini , E. Martins , I. Scurci , O. Hartley , J. Biol. Chem. 2018, 293, 19092–19100. 30305389 10.1074/jbc.RA118.004370 PMC6295741

[31] J. Bouchenna , M. Sénéchal , H. Drobecq , N. Stankovic-Valentin , J. Vicogne , O. Melnyk , Bioconjugate Chem. 2019, 30, 2684–2696. 10.1021/acs.bioconjchem.9b00598 31532181

[32] G. M. Fang , Y. M. Li , F. Shen , Y. C. Huang , J. Bin Li , Y. Lin , H. K. Cui , L. Liu , Angew. Chem. Int. Ed. 2011, 50, 7645–7649; 10.1002/anie.201100996 21648030

[33] R. E. Thompson , X. Liu , N. Alonso-García , P. J. B. Pereira , K. A. Jolliffe , R. J. Payne , J. Am. Chem. Soc. 2014, 23, 8161–8164. 10.1021/ja502806r 24873761

[34] Q. Wan , S. J. Danishefsky , Angew. Chem. Int. Ed. 2007, 46, 9248–9252; 10.1002/anie.200704195 18046687

[35] D. Mukhopadhyay , F. Ayaydin , N. Kolli , S. H. Tan , T. Anan , A. Kametaka , Y. Azuma , K. D. Wilkinson , M. Dasso , J. Cell Biol. 2006, 174, 939–949. 17000875 10.1083/jcb.200510103 PMC2064386

[36] C. Maison , K. Romeo , D. Bailly , M. Dubarry , J. P. Quivy , G. Almouzni , Nat. Struct. Mol. Biol. 2012, 19, 458–460. 22388734 10.1038/nsmb.2244

[37] F. Liebelt , N. S. Jansen , S. Kumar , E. Gracheva , L. A. Claessens , M. Verlaan-de Vries , E. Willemstein , A. C. O. Vertegaal , Nat. Commun. 2019, 10, 1–18. 31485003 10.1038/s41467-019-11773-x PMC6726658

[38] N. Hattersley , L. Shen , E. G. Jaffray , R. T. Hay , Mol. Biol. Cell 2011, 22, 78–90. 21148299 10.1091/mbc.E10-06-0504 PMC3016979

[39] Y. Wang , C. Chen , X. Meng , J. Fan , M. Pan , J. Chen , H. Deng , J. Shi , L. Liu , Y. M. Li , CCS 2021, 3, 1157–1168.

[40] J. Bouchenna , M. Sénéchal , H. Drobecq , J. Vicogne , O. Melnyk , Total Bioconjugate Chem. 2019, 30, 2967–2973. 10.1021/acs.bioconjchem.9b00661 31702897

