# Supplementary material for: In‐Plate Chemical Synthesis of Isopeptide‐Linked SUMOylated Peptide Fluorescence Polarization Reagents for High‐Throughput Screening of SENP Preferences
Source: Chembiochem. 2022 Dec 7;24(2):e202200601. doi: 10.1002/cbic.202200601 (PMC10107784; doi:10.1002/cbic.202200601)
Supplement: Supplementary file 1 — Supporting Information [file CBIC-24-0-s001.pdf]

ChemBioChem

Supporting Information

In-Plate Chemical Synthesis of Isopeptide-Linked SUMOylated Peptide Fluorescence Polarization Reagents for High-Throughput Screening of SENP Preferences

Yara Huppelschoten, Rishov Mukhopadhyay, Jens Buchardt, Thomas E. Nielsen, Alfred C. O. Vertegaal, Huib Ovaa[#], and Gerbrand J. van der Heden van Noort*

General procedures

Materials and solvents

Reagents were obtained from Sigma-Aldrich of the highest available grade and used without further purification. Standard Fmoc-protected amino acid derivatives were used and purchased from Gyros Protein Technologies unless mentioned otherwise. Fmoc-Cys(Acm)-OH and resins for SPPS were obtained from Novabiochem (Merck Millipore), Apigenex and PCAS Biomatrix. Pseudoproline dipeptides were obtained from Corden Pharma or Bachem. Iso-acyl dipeptides were obtained from AAPPTec. Solvents for SPPS were obtained from Biosolve. VA-044 was procured from Wako Pure Chemical Corporation. Oxyma Pure® was purchased from Gyros Protein Technologies. HPLC grade acetonitrile was obtained from Merck.

Analytical methods

LC-MS conditions

LC-MS measurements were performed on a Waters Acquity UPLC H Class system, Waters Xevo G2-XS QTof with a Waters Acquity BEH 300 Å, C4, 1.7 µm, 2.1 mm x 50 mm (0.4 mL/min). Samples were run at 60 °C using 3 mobile phases: A = 0.1 % formic acid in MilliQ water, B = 0.1 % formic acid in acetonitrile and C = 0.01 % TFA in MilliQ water with a gradient of 5 to 25% B over 1 min, 25 to 65 % B over 6 min followed by 65 to 95 % B over 0.5 min maintaining a composition of 5% C throughout. Data processing was performed using Waters MassLynx Mass Spectrometry Software V4.2 (deconvolution with MaxEnt I function).

UPLC conditions

UPLC measurements were performed on a Waters Acquity UPLC H Class system with a Waters Acquity BEH column, C18, 1.7 µm, 2.1 mm x 50 mm (0.4 mL/min). Samples were run at 40 °C using 2 mobile phases: A = 0.05 % TFA in MilliQ water and B = 0.05 % TFA in acetonitrile with a gradient of 5 to 60 % B over 3.5 min followed 60 to 100% B over 0.5 min. Data processing was performed using Empower software.

Quantification

Charged Aerosol Detection (CAD)

Purified samples were quantified using a Thermo Scientific Vanquish, Corona Veo CAD. Samples were run Acquity BEH 300 Å, C4, 1.7 µm, 2.1 mm x 50 mm at 40 °C using 2 mobile phases: A = 0.1 % TFA in MilliQ water and B = 0.1 % TFA in acetonitrile with a gradient of 0 to 80 % B over 7 min.

Protein expression and purification

The human catalytic domains of SENP1 (amino acids 419-644), SENP2 (amino acids 364-589), SENP5 (amino acids 568-755), SENP6 (amino acids 628-1112) and SENP7 (amino acids 706-1050) were cloned into a pET28a vector (Addgene plasmid # 16357). All constructs encoded an N-terminal 6xHis-tag followed by a Thrombin cleavage site. Overexpression was performed in *Escherichia coli* BL21-CodonPlus (DE3)-RIL strain and grown in LB media using a LEX bioreactor or a shaker incubator. Cells were allowed to grow at 37 ˚C until an OD_{600} of 1.2. Following this, protein expression was induced with 0.3 mM isopropyl– β-D thiogalactopyranoside (IPTG). Induction for SENP 1, 2 and 5 was performed at 30 ˚C for 3 hours and for SENP6 and 7 at 25 ˚C for 5 hours. Cells were harvested and resuspended in lysis buffer (50 mM HEPES, 200 mM NaCl and 5% glycerol). Buffer was supplemented with phenylmethylsulfonylfluoride (PMSF) (1 mM) and benzamidine (1 mM). Lysis was performed using a sonicator 3000; misonix for 3 minutes with 15-/45-s on/off cycles followed by centrifugation at 24,000 x g for 45 minutes at 4°C. The proteins were purified by his-tag purification using Qiagen Ni-NTA resin. The beads were washed with washing buffer (50 mM HEPES, 200 mM NaCl, 20 mM

Imidazole) and eluted with elution buffer (50 mM HEPES, 200 mM NaCl, 200 mM Imidazole and 2 mM TCEP). Thereafter, proteins were dialyzed overnight in buffer containing 20 mM HEPES, 100 mM NaCl, 2 mM TCEP. After dialysis the proteins were further purified using size exclusion in 20 mM HEPES, 100 mM NaCl and 2 mM TCEP using a superdex 75 10/300 GL column (GE Healthcare). Fractions were pooled and concentrated using amicon spin filters with a MW cut off of 30 kDa. The concentrated fractions were flash frozen and stored at -80 ˚C until further use. All SENPs expressed with an average yield between 1-5 mg/L.

Solid Phase Peptide Synthesis (SPPS)

Automated Fmoc SPPS

Method 1

SPPS was performed on a Symphony X (Gyros Protein Technologies) automated peptide synthesizer using standard 9-fluorenylmethoxycarbonyl (Fmoc) based SPPS. Fmoc deprotection was achieved with 2 x 10 min. treatment of 20 vol. % piperidine, 0.1 % Oxyma Pure® in DMF. Peptide couplings were performed using DIC/Oxyma. Amino acid/Oxyma solutions (0.3 M/0.3 M in DMF) were added to the resin at 4-6-fold excess together with equal equivalents of DIC (1.5 M in DMF). The coupling time was 2 hours unless specified otherwise. All dipeptide building blocks were coupled for 4 hours. The residual free amino groups after the coupling reaction were capped by the addition of collidine (3.3 equiv., 1.5 M in DMF) and acetic anhydride (11 equiv., 1.0 M in DMF) and were reacted for 20 minutes. After the final Fmoc deprotection the resin was washed with DMF and DCM.

Method 2

SPPS was performed on a SYRO II (Multisyntech, SYRO Robot) automated peptide synthesizer using standard 9-fluorenylmethoxycarbonyl (Fmoc) based SPPS. Fmoc deprotection was achieved with 2 x 2 min. and 1x 5 min treatment of 20 vol. % piperidine in NMP. Peptide couplings were performed using PyBOP/DIPEA. Amino acid solutions (0.34 M in NMP) were added to the resin at 4-fold excess together with an 8-fold excess of DIPEA (1.36 M in NMP) and 8-fold excess of PyBOP (0.34 M in NMP). The coupling time was 2x 25 minutes unless specified otherwise. After the final Fmoc deprotection the resin was washed with NMP and DCM.

Synthesis in 96-well plate format

The synthesis was performed using an automated peptide synthesizer (Intavis, Multipep CF) using standard 9-fluorenylmethoxycarbonyl (Fmoc) based SPPS. Fmoc deprotection was achieved with 2 x 10 min. treatment of 20 vol. % piperidine, 0.1 % Oxyma Pure® in DMF. Peptide couplings were performed using DIC/Oxyma Pure®. Amino acid/Oxyma Pure® solutions (0.3 M/0.3 M in DMF) were added to the resin at 4-fold excess together with equal equivalents of DIC (1.5 M in DMF). Triple couplings were performed with a coupling time of 1 hour. After the final Fmoc deprotection the resin was washed with DMF and DCM.

Global deprotection from the resin and side chain deprotection

Polypeptide sequences containing a cysteine residue were detached from the resin and deprotected by treatment with Reagent K (TFA/phenol/H_2O/thioanisole/EDT, 82.5:5:5:5:2.5 v/v/v/v/v) for 2-3 hours followed by precipitation in ice cold diethylether and collection by centrifugation. The pellet was resuspended in diethylether before being collected by centrifugation again. The pellet was dissolved in H_2O/CH_3CN/AcOH, 65:25:10, v/v/v and lyophilized before purification.

Purification of peptides

Preparative purification was performed on a Gilson HPLC system using a Phenomenex, Luna 100 Å, C8(2), 10 µm, 30 mm x 250 mm column. Elution was performed using 2 mobile phases: A = 0.1 % TFA in MilliQ water and B = 0.1% TFA in acetonitrile using a linear gradient. Fractions were collected using

a Gilson fraction collector and relevant fractions were assessed by analytical LC-MS. Fractions containing the pure peptide were pooled and lyophilized.

Semi-preparative purification was performed on an Agilent 1260 Infinity II using fluorescence detection. Elution was performed using 2 mobile phases: A = 0.1 % TFA in MilliQ water and B = 0.1% TFA in acetonitrile using a linear gradient. Fractions were assessed by analytical LC-MS. Fractions containing the pure peptide were pooled and lyophilized.

Table S1. Amino acids sequences of all peptides and their corresponding position in the 96-well plate derived protein and position in the protein.

#	Well	Protein	Lysine	Seqeunce	#	Well	Protein	Lysine	Seqeunce
**1**	A 1	TRIM28	779	KLTEDKADVQS	**49**	E 1	ZNF106	911	ATMHLKQEVTP
**2**	A 2	SUMO2	11	PKEGVKTENND	**50**	E 2	TOP1	117	SPPQIKDEPED
**3**	A 3	PML	490	PRKVIKMESEE	**51**	E 3	XRCC6	556	GSKRPKVEYSE
**4**	A 4	RANGAP1	524	HMGLLKSEDKV	**52**	E 4	SPRTN	423	DNFFIKKEQIK
**5**	A 5	RSF1	277	EETTVKKEKED	**53**	E 5	SART1	709	YKPDVKIEYVD
**6**	A 6	TRIM28	750	ARLQEKLSPPY	**54**	E 6	MKI67	2734	QKVQVKEEPSA
**7**	A 7	HNRNPM	698	GCGVVKFESPE	**55**	E 7	IFI16	561	LKPRLKTEPEE
**8**	A 8	SAFB2	293	LLAVVKREPAE	**56**	E 8	TDG	330	KKMAVKEEKYD
**9**	A 9	SUMO3	11	PKEGVKTENDH	**57**	E 9	PIAS1	238	YLPPTKNGVEP
**10**	A10	SNIP1	30	AGVVVKQERLS	**58**	E10	UBC	48	LIFAGKQLEDG
**11**	A11	TOP2A	1240	NKKKIKNENTE	**59**	E11	MKI67	1035	GKVGVKEELLA
**12**	A12	SART1	141	EVNAIKKEAGT	**60**	E12	PIAS1	315	ALIKEKLTADP
**13**	B 1	TRIM33	776	DQVKVKQEPGT	**61**	F 1	SUMO2	7	ADEKPKEGVKT
**14**	B 2	NOP58	467	AKIKVKVEEEE	**62**	F 2	KIF23	662	IVTEPKTEKPE
**15**	B 3	GTF2I	221	GPIKVKTEPTE	**63**	F 3	SP100	297	RLVDIKKEKPF
**16**	B 4	GTF2I	488	GSTEAKAVPYQ	**64**	F 4	BAZ1B	826	KKEIVKFEPQV
**17**	B 5	NPM1	32	KDYHFKVDNDE	**65**	F 5	PBRM1	1293	IYYFRKPIVPQ
**18**	B 6	UBC	11	KTLTGKTITLE	**66**	F 6	ZNF106	1737	LVRIYKGHNHA
**19**	B 7	PARP1	486	WGAEVKAEPVE	**67**	F 7	CBX3	103	ESDDSKSKKKR
**20**	B 8	PML	160	HQWFLKHEARP	**68**	F 8	CBX4	280	QAVKIKSGEVA
**21**	B 9	HSF2	82	DSGIVKQERDG	**69**	F 9	MKI67	2009	GKVGVKEEVLP
**22**	B10	BCLAF1	831	FQKRPKEEEWD	**70**	F10	PARP1	467	VSASTKSLQEL
**23**	B11	BRD4	1111	PLVVVKEEKIH	**71**	F11	PIAS2	443	SQPCTKIESSS
**24**	B12	SAFB2	225	LGETCKSEPVK	**72**	F12	TOP2B	1227	KPKVKKLQLEE
**25**	C 1	TRIM24	723	EPIRIKQENSG	**73**	G 1	UIMC1	20	RNLEKKDVETT
**26**	C 2	ZMYM4	250	SNIRIKEEPLD	**74**	G 2	XPC	81	AKVTVKSENLK
**27**	C 3	ZMYM4	273	LLDKIKDEPDN	**75**	G 3	ZNF451	832	KSNLYKFTASA
**28**	C 4	ZBTB2	505	VLASIKKEQET	**76**	G 4	PARP1	203	QLPGVKSEGKR
**29**	C 5	HNRNPM	17	AATEIKMEEES	**77**	G 5	SUMO2	5	_MADEKPKEGV
**30**	C 6	SUMO1	7	SDQEAKPSTED	**78**	G 6	ZMYM2	98	QGNDSKITPSS
**31**	C 7	UBE2I	49	AIPGKKGTPWE	**79**	G 7	SUMO1	37	SEIHFKVKMTT
**32**	C 8	GTF2I	456	PASGVKEEWYA	**80**	G 8	UBC	63	DYNIQKESTLH
**33**	C 9	SUMO3	41	HTPLSKLMKAY	**81**	G 9	PCNA	164	VISCAKDGVKF
**34**	C10	RSF1	456	VAPNFKTEPIE	**82**	G10	PML	380	GFDEFKVRLQD
**35**	C11	SAFB2	517	HSVEIKIEKTV	**83**	G11	NOP56	540	KKSTPKEETVN
**36**	C12	GTF2I	991	GSSQIKQEPDP	**84**	G12	PIAS2	249	YAPPPKNGIEQ
**37**	D 1	ZBTB21	879	LKIQVKEEPVE	**85**	H 1	SP100	306	PFSNSKVECQA
**38**	D 2	ZNF451	706	TETSIKTEDDF	**86**	H 2	PIAS1	46	ALHLLKAGCSP
**39**	D 3	NUMA1	1766	QRLPPKVESLE	**87**	H 3	PIAS1	137	VHPDIKLQKLP
**40**	D 4	BCLAF1	676	EERVFKEENQK	**88**	H 4	ETV6	11	AQCSIKQERIS
**41**	D 5	PARP1	512	SKGQVKEEGIN	**89**	H 5	HNRNPM	388	GEIIAKQGGGG
**42**	D 6	ZNF451	130	NNSDTKISETE	**90**	H 6	SUMO1	17	DLGDKKEGEYI
**43**	D 7	SUMO3	32	SVVQFKIKRHT	**91**	H 7	TP53BP1	217	ANTAIKHEEQS
**44**	D 8	TOP1	153	KPKKIKTEDTK	**92**	H 8	TP53BP1	1434	LSPDDKSFSRV
**45**	D 9	CCAR1	1012	PTPTVKQESKD	**93**	H 9	TP53BP1	1563	VKGHRKESGEL
**46**	D10	TOP2A	1228	ITIEMKAEAEK	**94**	H10	RANBP2	2571	SGSESKVEPKK
**47**	D11	ADAR	418	QEPVIKLENRQ	**95**	H11	CDK11B	243	GRKPVKEEKME
**48**	D12	TRIM33	793	FSGGVKQEKTE	**96**	H12	MCM3	732	DSQETKESQKV

Synthesis of SUMO3 thioester (1)

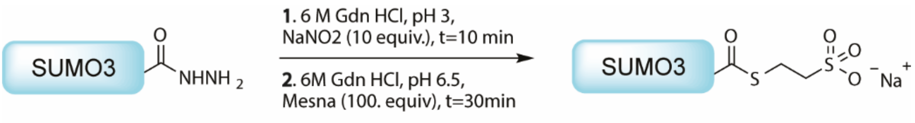

1

The synthesis was performed following general procedures (method 1) using 2-chlorotrityl hydrazine resin (0.32 mmol/gram). The peptide was cleaved from the resin according to the general procedures and the crude peptide was directly used for thioesterification. Crude peptide **1** (215 mg, 0.02 mmol) was dissolved in 12 mL of 6 M Gdn.HCl pH 3.0 and 1 M $NaNO_2$ in MilliQ (0.2 mL, 0.2 mmol, 10 equiv.) was added and stirred for 15 min at 0 °C. The reaction was warmed to room temperature and MESNa (259 mg, 1.5 mmol, 75 equiv.) in 2 mL of 6 M Gdn.HCl, 0.2 M phosphate pH 7.0 was added. The pH was adjusted to pH 7.0 and the solution was stirred for 30 min. before purification by preparative RP-HPLC using Phenomenex, Luna 100 Å, C8(2), 10 µm, 30 mm x 250 mm (25 to 35 % B over 30 min, 30mL/min) followed by lyophilization afforded peptide **1** as a white solid (21 mg, 0.002 mmol 10 % yield).

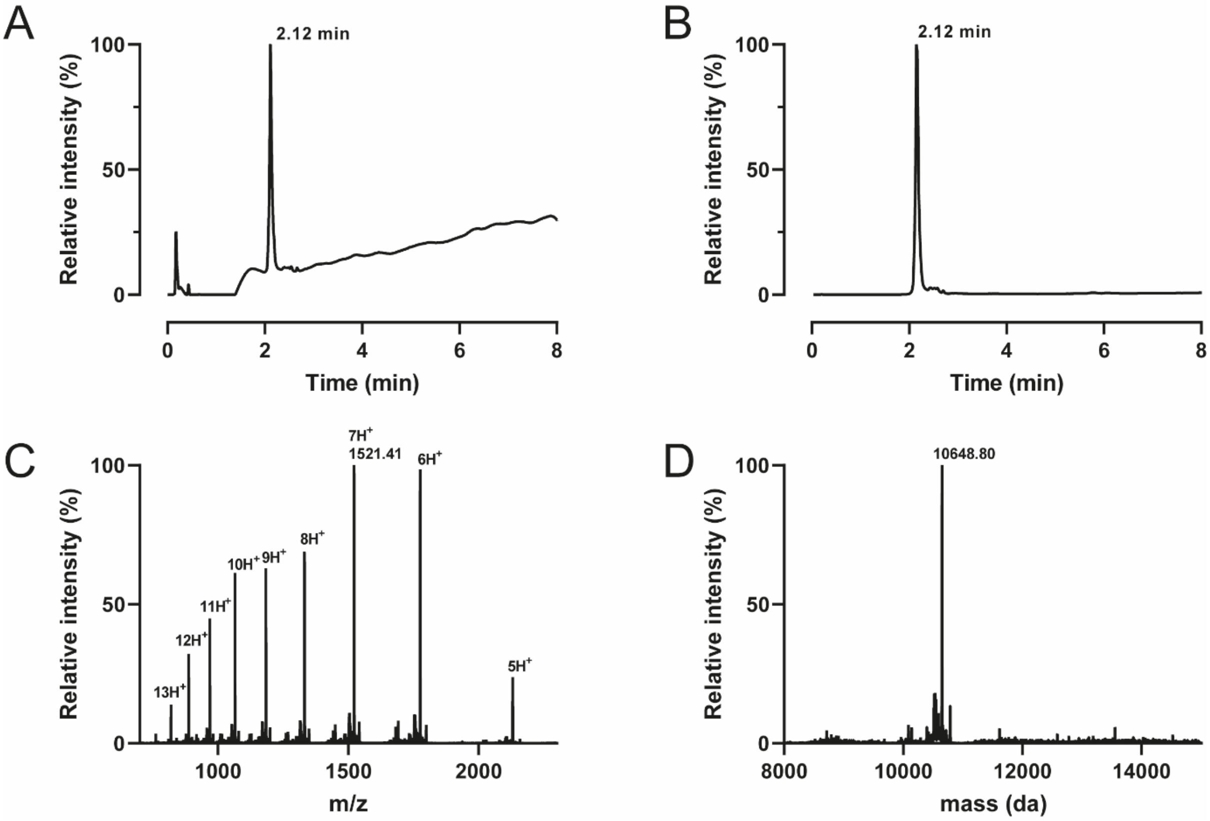

Figure S1. A. UV chromatogram of purified **1**, Rt 2.12 min. **B.** Total ion spectrum of purified **1**, Rt 2.12 min. **C.** Observed ESI spectrum of purified **1**. Calculated Mass (average isotope composition): 10642.16; $[M + 5H]^{5+}$: 2129.43, $[M + 6H]^{6+}$: 1774.69, $[M + 7H]^{7+}$: 1521.31, $[M + 8H]^{8+}$: 1331.27, $[M + 9H]^{9+}$: 1183.46, $[M + 10H]^{10+}$: 1065.22, $[M + 11H]^{11+}$: 968.47, $[M + 12H]^{12+}$: 887.85, $[M + 13H]^{13+}$: 819.63. Observed; $[M + 5H]^{5+}$: 2129.56, $[M + 6H]^{6+}$: 1774.81, $[M + 7H]^{7+}$: 1521.41, $[M + 8H]^{8+}$: 1331.36, $[M + 9H]^{9+}$: 1183.55, $[M + 10H]^{10+}$: 1065.31, $[M + 11H]^{11+}$: 968.55, $[M + 12H]^{12+}$: 887.93, $[M + 13H]^{13+}$: 819.70. **D.** Deconvoluted mass of purified **1**, mass calculated: 10648.9, observed: 10648.80.

Synthesis of RANGAP1 (A4)

The synthesis was performed following general procedures (method 1) using Rink Amide resin (446 mg, 0.59 mmol/gram). The peptide was cleaved from the resin using TFA/H_2O/TIPS 95:2.5:2.5, v/v/v followed by evaporation of the TFA using nitrogen flow. Next, the peptide was dissolved in a mixture of H_2O/ACN 90:10, v/v, followed by purification by preparative RP-HPLC using a Phenomenex, Gemini® 110 Å, C18, 5 µm, 30 mm x 250 mm column (10 to 30 % B over 25 min, 30mL/min). The fractions were analyzed and pooled according to purity followed by lyophilization afforded RANGAP1 as a pink solid (71 mg, 0.037 mmol 14 % yield).

Synthesis of 96-well plate containing peptides

The synthesis was performed following general procedures using 8 µM of Rink Amide resin (100-200 mesh, Novabiochem, HL, 057 mmol/gram) per well. Ass final coupling Fmoc-PEG_2-OH and TAMRA were coupled following general procedures method to all peptides. Next, the peptides were cleaved from the resin using TFA/H_2O/TIPS 95:2.5:2.5, v/v/v followed by evaporation of the TFA. Peptides were dissolved in a mixture of H_2O/ACN 90:10, v/v, and purified using a Waters Arc HPLC system containing a Waters ACQUITY QDa Detector, fraction collector and a Phenomenex, Gemini® 110 Å, C18, 5 µm, 10 mm x 150 mm column. Fractions were collected based on molecular weight, analyzed using UPLC and pooled based on purity before lyophilization. Next, the peptides were dissolved in 500 µL of a H_2O/ACN 90:10 mixture and the concentration was determined using CAD as described in general procedures to determine the amount of peptide (table S2). 95 peptides were obtained with an average yield of 3.4 % ranging from 0.5 % to 8.5 %.

Table S2. Calculated yields per peptide. Peptide in red failed during synthesis or purification.

Well	MW	Mg obtained	Yield (%)	Well	MW	Mg obtained	Yield (%)
A 1	1909.53	0.13	0.82	E 1	1930.53	0.63	4.09
A 2	1906.43	0.53	3.46	E 2	1930.43	0.38	2.46
A 3	2021.73	1.38	8.52	E 3	1955.63	0.53	3.39
A 4	1932.73	N.D.	N.D.	E 4	2085.93	0.9	5.39
A 5	2011.53	0.19	1.16	E 5	2044.73	0.75	4.59
A 6	1977.73	0.97	6.16	E 6	1918.43	0.15	1
A 7	1827.43	0.24	1.65	E 7	2015.73	0.36	2.24
A 8	1900.63	1.02	6.7	E 8	2044.83	0.59	3.63
A 9	1929.43	0.61	3.97	E 9	1890.53	0.18	1.2
A10	1861.53	0.8	5.37	E10	1866.73	0.78	5.21
A11	2021.73	0.1	0.64	E11	1818.63	0.61	4.2
A12	1835.53	0.62	4.19	E12	1874.73	0.32	2.14
B 1	1904.43	0.51	3.36	F 1	1877.53	1.09	7.28
B 2	1977.63	0.23	1.48	F 2	1946.53	1.05	6.74
B 3	1874.53	0.86	5.75	F 3	2048.93	1.05	6.4
B 4	1826.43	0.35	2.39	F 4	2020.73	0.63	3.88
B 5	2085.63	0.2	1.17	F 5	2099.93	0.61	3.65
B 6	1880.73	0.66	4.37	F 6	1983.73	0.84	5.3
B 7	1890.43	0.59	3.92	F 7	1983.73	0.58	3.67
B 8	2124.73	0.4	2.36	F 8	1805.53	0.26	1.8
B 9	1879.53	0.53	3.54	F 9	1830.53	0.47	3.19
B10	2167.73	0.42	2.41	F10	1838.53	0.61	4.17
B11	1966.63	0.45	2.88	F11	1842.43	0.07	0.49
B12	1866.53	0.38	2.51	F12	2015.83	0.23	1.45
C 1	1946.63	0.29	1.83	G 1	2008.63	0.29	1.8
C 2	1989.73	0.63	3.96	G 2	1892.63	0.27	1.78
C 3	1975.73	0.86	5.47	G 3	1905.73	0.51	3.35
C 4	1921.63	0.89	5.77	G 4	1874.63	0.28	1.87
C 5	1913.53	0.13	0.86	G 5	1779.43	1.2	8.46
C 6	1882.33	0.46	3.06	G 6	1809.43	0.5	3.47
C 7	1859.63	1.03	6.92	G 7	1996.73	1.07	6.72
C 8	1912.53	0.68	4.43	G 8	2023.63	0.55	3.37
C 9	1964.83	0.61	3.91	G 9	1842.63	0.15	1
C10	1920.53	0.71	4.64	G10	2029.73	0.71	4.34
C11	1958.63	0.44	2.81	G11	1936.53	0.44	2.85
C12	1861.43	0.46	3.09	G12	1889.53	0.37	2.47
D 1	1987.63	0.86	5.41	H 1	1885.43	0.09	0.57
D 2	1961.53	0.82	5.25	H 2	1785.63	0.14	0.97
D 3	1971.63	0.81	5.15	H 3	1963.73	0.79	5.01
D 4	2111.63	0.21	1.22	H 4	1938.63	N.A.	N.A.
D 5	1864.53	0.38	2.52	H 5	1662.53	0.42	3.16
D 6	1913.43	0.11	0.69	H 6	1942.73	0.37	2.41
D 7	2018.73	0.67	4.17	H 7	1903.43	0.12	0.82
D 8	1991.83	0.34	2.12	H 8	1926.63	0.4	2.61
D 9	1905.43	0.11	0.7	H 9	1915.63	0.54	3.49
D10	1938.73	0.76	4.88	H10	1851.53	0.44	2.99
D11	2029.63	0.36	2.23	H11	2006.73	0.58	3.62
D12	1885.53	0.45	2.99	H12	1954.43	0.48	3.07

Table S3. Calculated and observed molecular weights after NCL and desulfurization. Peptides in orange are double/non- desulfurized. Peptides in red were produced in non-quantifiable amounts and omitted from the FP-screen.

Well	MW calculated	MW found	MW cal desulfurized	MW found	Yield (%)	Well	MW calculated	MW found	MW cal desulfurized	MW found	Yield (%)
A 1	12328.6	12329.2	12297.2	12296.4	1.52	E 1	12349.6	12350.8	12318.8	12320.2	1.28
A 2	12325.5	12326.2	12294.2	12294	3.76	E 2	12349.5	12351.4	12319.4	12320.2	4.05
A 3	12440.8	12441.4	12408.8	12409	1.18	E 3	12374.7	12375.4	12343.4	12345	4.59
A 4	12351.8	12352.2	12320.2	12320	N.D.	E 4	12505	12505.6	12473.6	12475.6	2.57
A 5	12430.6	12431.2	12398.6	12398.8	0.81	E 5	12463.8	12465.4	12433.4	12431.8	1.39
A 6	12396.8	12397.4	12365.4	12365.2	2.39	E 6	12337.5	12338.8	12306.8	12308.4	1.78
A 7	12246.5	12247.4	12215.4	12182.6	2.64	E 7	12434.8	12436.2	12404.2	12405	4.6
A 8	12319.7	12320.2	12288.2	12288.2	N.A.	E 8	12463.9	12464.8	12432.8	12434.2	5.36
A 9	12348.5	12349.4	12317.4	12316.8	3.14	E 9	12309.6	12310.8	12278.8	12279.8	N.A.
A10	12280.6	12281.4	12249.4	12249	0.5	E10	12285.8	12286.2	12254.2	12256	4.17
A11	12440.8	12441.4	12409.4	12409	N.A.	E11	12237.7	12238.6	12206.6	12207.4	2.6
A12	12254.6	12255	12223	12223	4.5	E12	12293.8	12294.6	12262.6	12263.6	3.5
B 1	12323.5	12324.2	12292.2	12294	4.78	F 1	12296.6	12297.8	12265.8	12265	6.18
B 2	12396.7	12397	12365	12366.6	1.08	F 2	12365.6	12366.6	12334.6	12336	6.67
B 3	12293.6	12294	12262	12263.6	6.04	F 3	12468	12469	12437	12438.2	6.21
B 4	12245.5	12246.2	12214.2	12215.8	4.72	F 4	12439.8	12441	12409	12410	5.71
B 5	12504.7	12505.4	12473.4	12474.6	0.83	F 5	12519	12441.6	12409.6	12410.8	1.47
B 6	12299.8	12300.4	12268.4	12269.6	4.95	F 6	12402.8	12404.4	12372.4	12373.6	3.11
B 7	12309.5	12310.8	12278.8	12279.6	2.73	F 7	12402.8	12403.8	12371.8	12374	5.52
B 8	12543.8	12544.6	12512.6	12514.2	3.18	F 8	12224.6	12226.2	12194.2	12195.6	2.79
B 9	12298.6	12300	12268	12268.8	3.4	F 9	12249.6	12250.8	12218.8	12219.6	3.84
B10	12586.8	12587.4	12555.4	12556.8	3.02	F10	12257.6	12259	12227	12228.4	3.3
B11	12385.7	12386.2	12354.2	12355.2	3.53	F11	12261.5	12263	12231	12199.8	N.A.
B12	12285.6	12286.2	12254.2	12223.6	3.64	F12	12434.9	12437	12405	12405.4	1.44
C 1	12365.7	12366.4	12334.4	12336.2	4.74	G 1	12427.7	12429.2	12397.2	12398.2	2.53
C 2	12408.8	12409.4	12377.4	12378.8	6.04	G 2	12311.7	12314	12282	12282.6	1.77
C 3	12394.8	12396	12364	12365	4.46	G 3	12324.8	12325.8	12293.8	12295.2	2.34
C 4	12340.7	12342	12310	12310.8	4.88	G 4	12293.7	12295.2	12263.2	12265	2.58
C 5	12332.6	12333	12301	12301.8	1.21	G 5	12198.5	12198	12166	12166.4	2.44
C 6	12301.4	12302.8	12270.8	12272	4.74	G 6	12228.5	12229.8	12197.8	12199.6	2.63
C 7	12278.7	12279.2	12247.2	12248.6	1.29	G 7	12415.8	N.A.	12383.8	12399.6	0.35
C 8	12331.6	12332	12300	12301.4	2.71	G 8	12442.7	12443.8	12411.8	12414.2	2.2
C 9	12383.9	12385	12353	12353.6	1.81	G 9	12261.7	12262.4	12230.4	12200	1.21
C10	12339.6	12340.4	12308.4	12309.8	6.3	G10	12448.8	12449.6	12417.6	12419.4	1.75
C11	12377.7	12378.2	12346.2	12347	1.6	G11	12355.6	12357.2	12325.2	12326.4	2.4
C12	12280.5	12281.2	12249.2	12251	4.36	G12	12308.6	12310.6	12278.6	12280.4	2.87
D 1	12406.7	12407.6	12375.6	12377.2	5.64	H 1	12304.5	12306.2	12274.2	12244	N.A.
D 2	12380.6	12381	12349	12350.6	6.21	H 2	12204.7	12205.4	12173.4	12143.2	0.82
D 3	12390.7	12391.6	12359.6	12393.2	N.A.	H 3	12382.8	12384.2	12352.2	12353.8	3.09
D 4	12530.7	N.A.	12498.7	N.A.	N.A.	H 4	12357.7	N.A.	12325.7	N.A.	N.A.
D 5	12283.6	12284.4	12252.4	12254	3.37	H 5	12081.6	12083.2	12051.2	12052.8	3.69
D 6	12332.5	12333.4	12301.4	12303.2	N.A.	H 6	12361.8	12363	12331	12332	2.83
D 7	12437.8	12438.6	12406.6	12408.2	N.A.	H 7	12322.5	12324.4	12292.4	12294.2	N.A.
D 8	12410.9	12411.8	12379.8	12379	4.16	H 8	12345.7	12346.8	12314.8	12316.2	2.30
D 9	12324.5	12325.8	12293.8	12295.2	N.A.	H 9	12334.7	12336	12304	12305.4	2.03
D10	12357.8	12359.2	12327.2	12327.8	1.37	H10	12270.6	12272.4	12240.4	12241.8	1.35
D11	12448.7	12450	12418	12419.6	3.21	H11	12425.8	12428	12396	12397	4.45
D12	12304.6	12305.6	12273.6	12275	4.47	H12	12373.5	12376.2	12344.2	12345.4	5.18

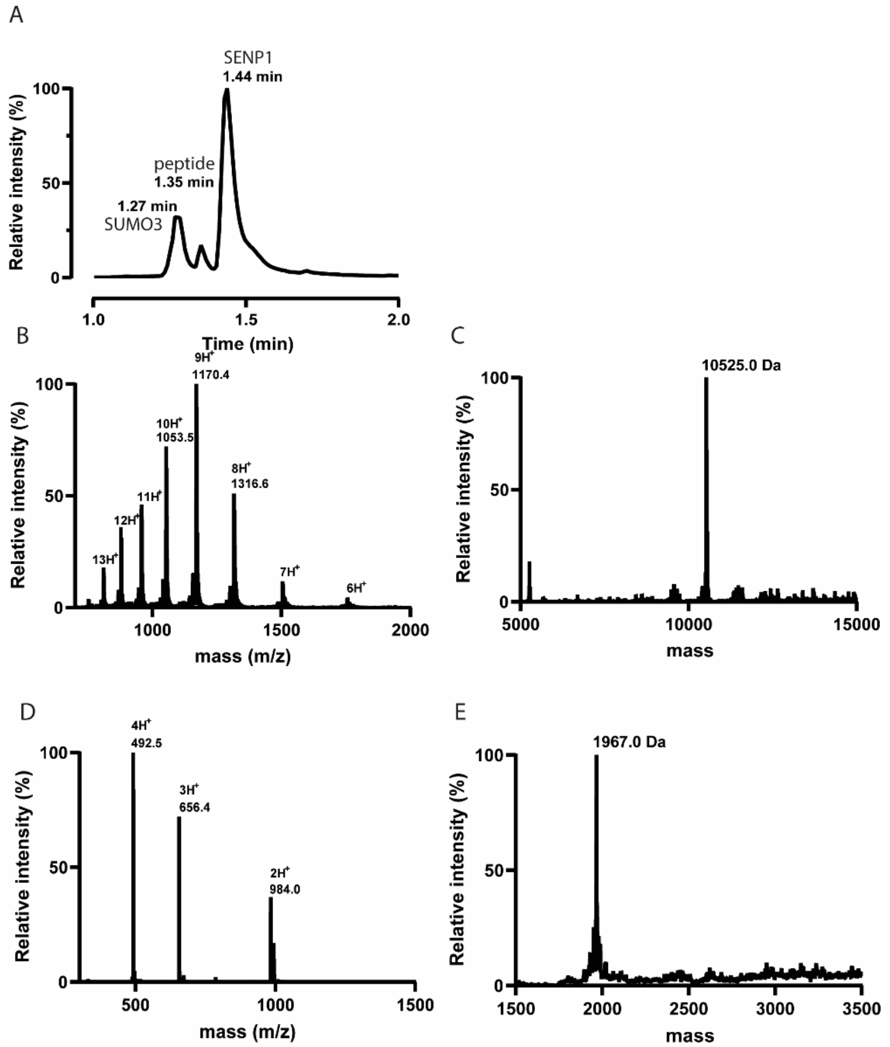

Figure S2. Determination of site of desulfurization on SUMO3-peptide conjugate E4. **A.** Total ion spectrum of E4 deconjugation by SENP1, **B.** ESI spectrum of SUMO3 (1.27 min). Calculated Mass (average isotope composition): $[M + 8H]^{8+}$: 1316.5, $[M + 9H]^{9+}$: 1170.4, $[M + 10H]^{10+}$: 1053.4, , observed: $[M + 8H]^{8+}$: 1316.5, $[M + 9H]^{9+}$: 1170.4, $[M + 10H]^{10+}$: 1053.5, **C.** Deconvoluted mass of SUMO3. Calculated: 10524.8, observed: 10525.0. **D.** ESI spectrum of peptide E4 (1.35 min). Calculated Mass (average isotope composition): $[M + 2H]^{2+}$: 984.5, $[M + 3H]^{3+}$: 656.7, $[M + 4H]^{4+}$: 492.8, Observed: $[M + 2H]^{2+}$: 984.0, $[M + 3H]^{3+}$: 656.4, $[M + 4H]^{4+}$: 492.5, **E.** Deconvoluted mass of peptide E4 corresponding to TMR-PEG2-DNFFIKKEQIK ; $C_{97}H_{135}N_{19}O_{25}$. Calculated: 1967.2, observed: 1967.0.

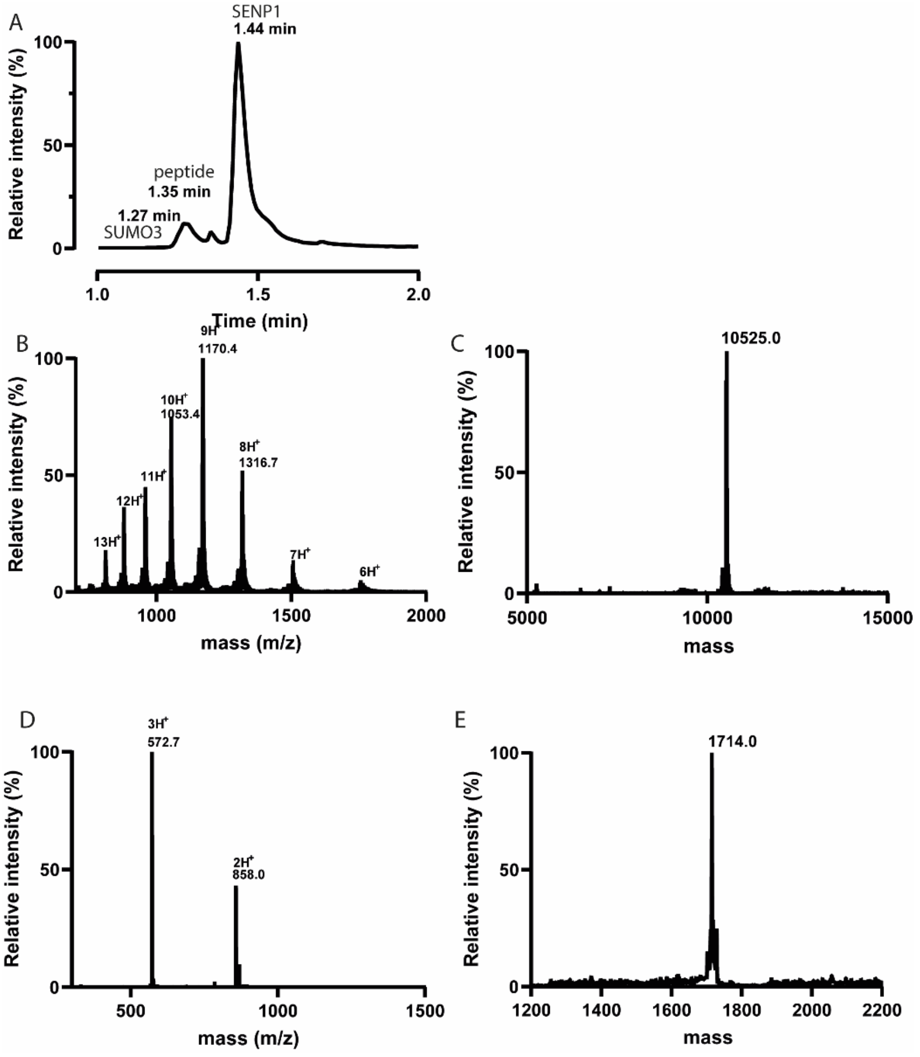

Figure S3. Determination of site of desulfurization on SUMO3-peptide conjugate B12. **A.** Total ion spectrum of B12 deconjugation by SENP1, **B.** ESI spectrum of SUMO3 (1.27 min). Calculated Mass (average isotope composition): $[M + 8H]^{8+}$: 1316.5, $[M + 9H]^{9+}$: 1170.4, $[M + 10H]^{10+}$: 1053.4, , observed: $[M + 8H]^{8+}$: 1316.7, $[M + 9H]^{9+}$: 1170.4, $[M + 10H]^{10+}$: 1053.4, **C.** Deconvoluted mass of SUMO3. Calculated: 10524.8, observed: 10525.0 **D.** ESI spectrum of peptide B12 (1.35 min). Calculated Mass (average isotope composition): $[M + 2H]^{2+}$: 858.4, $[M + 3H]^{3+}$: 572.6, Observed: $[M + 2H]^{2+}$: 858.0, $[M + 3H]^{3+}$: 572.7, **E.** Deconvoluted mass of peptide B12 corresponding to TMR-PEG2-LGETAKSEPVK ; $C_{81}H_{118}N_{16}O_{25}$. Calculated: 1715.9, observed: 1714.0.

Synthesis of TMR-K ^{SUMO3}G

TMR-K ^{SUMO3}G-was synthesized as previously described by Geurink et al. In short, peptide **1** (3.22 mg, 0.3 µmol) and TMR-thioKG (1.11 mg, 1.5 µmol) were dissolved in 6 M Gdn HCl, pH 7.4 (300 µL). Followed by the addition of Mesna and TCEP to a final concentration of 100 mM and 25 mM. The reaction was placed in a thermoblock/shaker at 37 °C and reacted for 16 hours. After LC-MS analysis showed that the reaction had reached completion, TCEP and VA-044 were added to a final concentration of 250 mM and 25 mM. The reaction was further incubated at 40 °C and allowed to react for 16 hours. The crude reaction mixture was purified by RP-HPLC using a Phenomenex, Luna 100 Å, C8(2), 10 µm, 30 mm x 250 mm column (18-40 % B over 20 min, flow 30 mL/min) and lyophilized to afford the desired peptide as a pink solid (0.8 mg, 24.0 yield%).

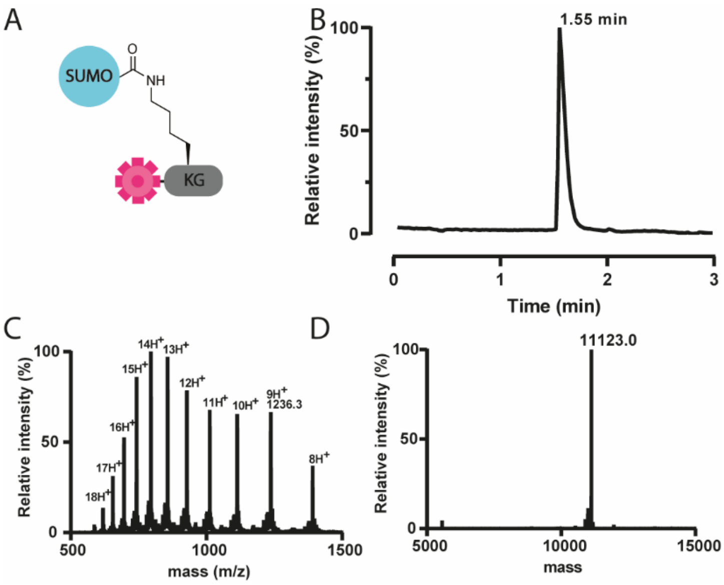

Figure S4. A. Schematic representation of TMR-K ^{SUMO3}G. **B.** Total ion spectrum of SUMO3 TMRKG, Rt 1.55 min. **C.** ESI spectrum of purified SUMO3 K48 TMRKG. Calculated Mass (average isotope composition): 11115.46; $[M + 7H]^{7+}$: 1588.92, $[M + 8H]^{8+}$: 1390.43, $[M + 9H]^{9+}$: 1236.05, $[M + 10H]^{10+}$: 1112.55, $[M + 11H]^{11+}$: 1011.50, $[M + 12H]^{12+}$: 927.28, $[M + 13H]^{13+}$: 856.04. Observed: 11117.6; $[M + 7H]^{7+}$: 1589.23, $[M + 8H]^{8+}$: 1390.5, $[M + 9H]^{9+}$: 1236.29, $[M + 10H]^{10+}$: 1112.57, $[M + 11H]^{11+}$: 1011.58, $[M + 12H]^{12+}$: 927.55, $[M + 13H]^{13+}$: 856.12. **D.** Deconvoluted mass of SUMO3 TMRKG. Calculated: 11122.4, observed: 11123.0.

Assembly of the SUMO probes

Peptides were dissolved in 1M TCEP (10% of the final volume) and incubated at RT for 30 minutes to remove the StBu protection group from the γ-thiolysine prior to the NCL. Thereafter, followed by the addition of SUMO3 thioester (90 % of the final volume) in 0.2 M Na_2HPO_4, 250 mM MESNa and 0.15 M NaCl pH 7.95 buffer, leading to a final concentration of 1 mM SUMO3. The NCL was shaken at 37 °C for 16 hours before LC-MS analysis was taken of all reactions. Followed by the addition of an equal amount of 0.2 M Na_2HPO_4, 500 mM TCEP and 50 mM VA-044 in MilliQ, resulting in a final buffer concentration of 0.2 M Na_2HPO_4, 250 mM TCEP, 125 mM MESNa and 25 mM VA-044 in MilliQ. The reaction was shaken at 350 rpm at 40 °C for 16 hours before LC-MS analysis was taken of all reactions. The volumes of each reaction were adjusted to 1 mL per well before purification by semi-preparative HPLC using a Phenomenex, Aeris™ widepore 200 Å, C4, 3.6 µm, 4.6 x 150 mm column followed by lyophilization afforded 83 peptides as pink solids with yields ranging from 0.2 % to 6.6 % with an average yield of 3.2 %.

Table S4. Final concentrations of SENPs in the FP assay.

Enzyme	*SENP1*	*SENP2*	*SENP5*	*SENP6*	*SENP7*
Concentration	20 pM	30 pM	200 pM	2.5 nM	2 nM

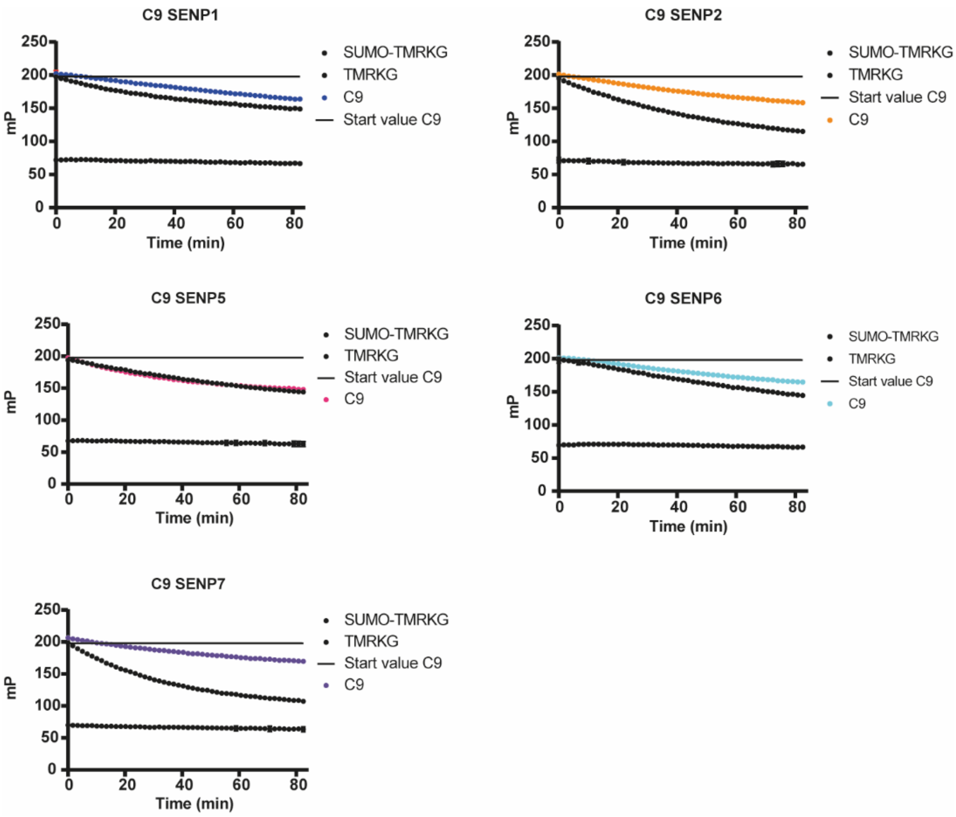

Figure S5. Reaction time-course of probe **C9** with SENP1, SENP2, SENP5, SENP6 and SENP7.

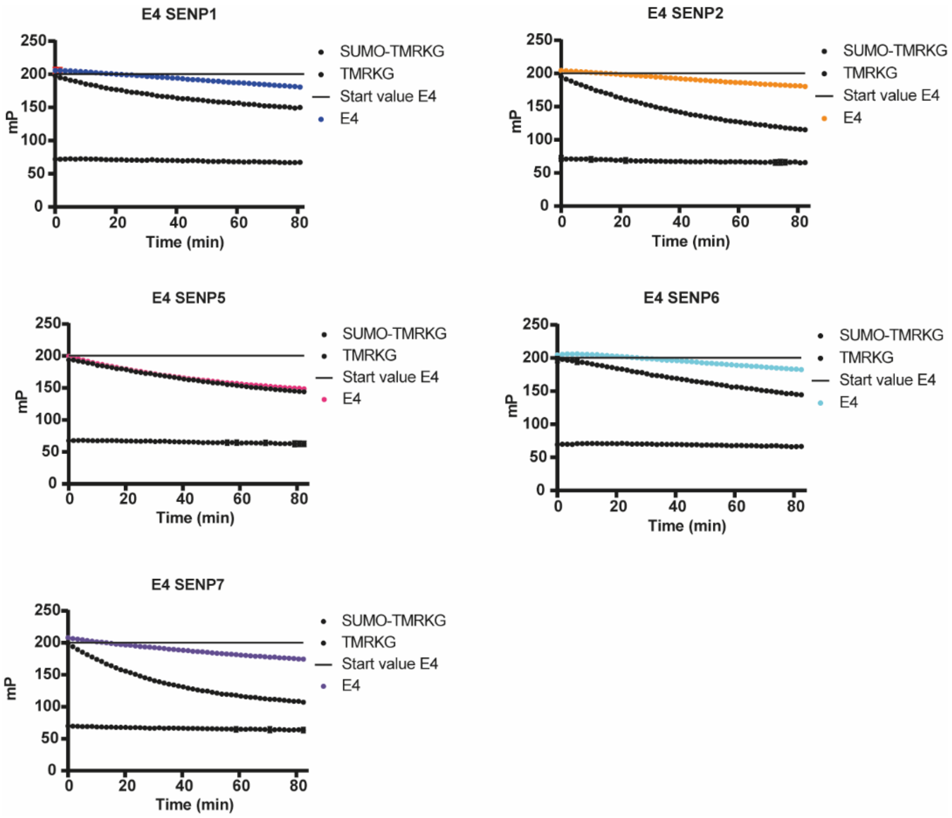

Figure S6. Reaction time-course of probe **E4** with SENP1, SENP2, SENP5, SENP6 and SENP7.

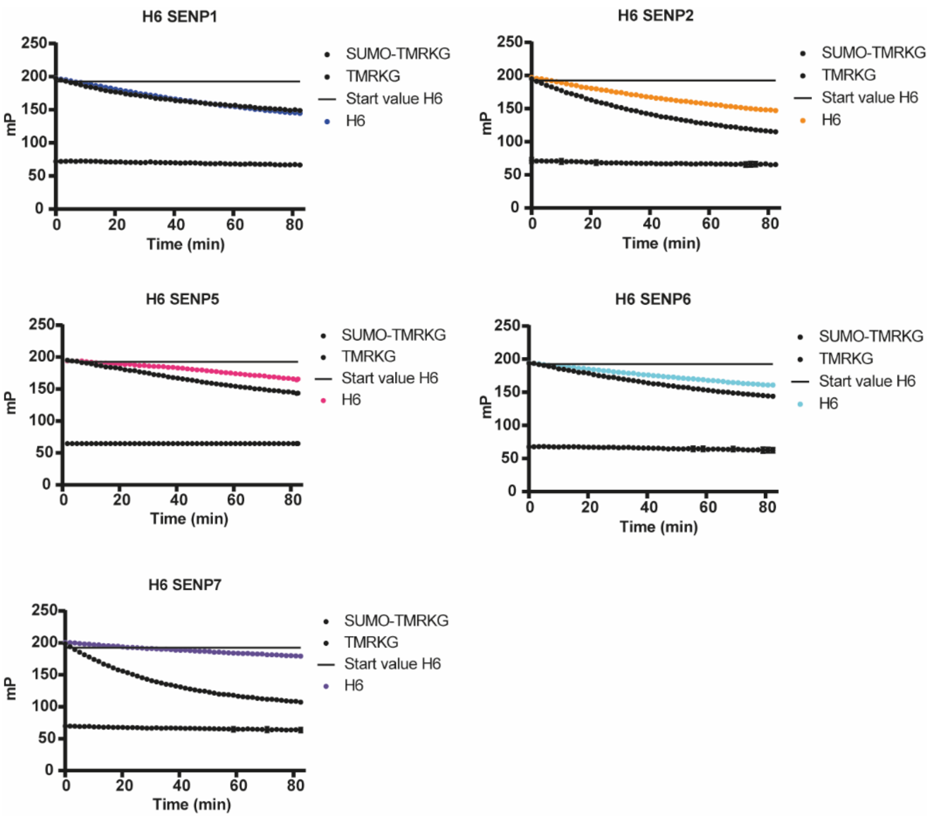

Figure S7. Reaction time-course of probe **H6** with SENP1, SENP2, SENP5, SENP6 and SENP7.

Table S5. Normalized data of the FP assay in %.

	SENP1	SENP2	SENP5	SENP6	SENP7
TRIM28$_{774-784}$	56	38,2	41,5	1	6,9
SUMO2$_{6-16}$	40,2	41	45,7	5,8	16,8
PML$_{485-495}$	56,8	41,1	72,5	17,5	27,6
RANGAP1$_{519-529}$	57,3	37,6	53,3	2,9	6,4
RSF1$_{272-282}$	18,5	29,2	34,8	3,1	12,7
TRIM28$_{745-755}$	78,6	50,1	93	30,2	18,5
HNRNPM$_{693-703}$	78,6	51,6	79,9	20,2	28,1
SUMO3$_{6-16}$	14,2	24,9	31,7	6,2	17,2
SNIP1$_{25-35}$	53,6	32,2	83,9	36,6	24,4
SART1$_{136-146}$	72,7	51,4	79,7	21,4	22,7
TRIM33$_{771-781}$	72,5	48,8	93,5	20,9	23,4
GTF2I$_{216-226}$	73,5	47,8	92	19	25
GTF2I$_{843-493}$	57,4	41,2	75	24,2	25,4
NPM1$_{27-37}$	46,9	36,6	48,4	9,6	18,3
UBC$_{6-16}$	76,8	48,5	101,9	35,5	29,1
PARP1$_{481-491}$	90,9	49,1	54,9	5,2	12,5
PML$_{155-165}$	89,4	48,9	100,7	42,5	25,6
HSF2$_{77-87}$	75,2	57,9	92,7	13,8	27,9
BCLAF1$_{826-836}$	70,1	43,6	50,9	10	16,6
BRD4$_{1106-1116}$	76,4	47,1	79,6	16,2	18,8
SAFB2$_{220-230}$	85,3	51,2	76,5	10,7	17,4

	SENP1	SENP2	SENP5	SENP6	SENP7
TRIM24$_{718-728}$	86,8	53,7	97,9	16,2	19,8
ZMYM4$_{245-255}$	83,3	49,5	83,2	8,6	21,6
ZMYM4$_{268-278}$	46	28,7	46,4	6,5	15,8
ZBTB2$_{500-510}$	101	60,9	107,3	26,6	21,7
HNRNPM$_{12-22}$	78,2	45,1	48,1	6,8	12,3
SUMO1$_{2-12}$	46,2	47,5	40,9	2,9	14,7
UBE2I$_{34-44}$	64,7	41,8	69,4	26,3	19,8
GTF2I$_{451-461}$	64,2	38,6	47	12,3	13,2
SUMO3$_{36-46}$	70,2	49,3	100,2	62,4	30,8
RSF1$_{451461}$	84,7	48,6	73,9	8,5	15,5
SAFB2$_{512-522}$	61,5	46,9	59,2	11	9,1
GTF2I$_{986-996}$	52,2	40,4	57,7	8,3	15
ZBTB21$_{874-884}$	76,8	50,2	68,9	9,5	18,1
ZNF451$_{701-711}$	69,7	45,2	47,3	0	9,1
PARP1$_{507-517}$	60,4	51	64,5	9,9	19,5
TOP1$_{148-158}$	12	15	33,7	24,4	29
TOP2A$_{1223-1233}$	82,8	44	65	14,9	13,3
ADAR$_{413-423}$	72,3	43,1	65,8	14,2	18,4
TRIM33$_{788-798}$	78,8	59,2	82,4	19,2	17,9

Color scale: 100 % / 50 / 0

	SENP1	SENP2	SENP5	SENP6	SENP7
ZNF106$_{906-916}$	86,4	51,7	84	15,7	20,7
TOP1$_{112-122}$	75,2	53	51,6	0,6	13,8
XRCC6$_{551-561}$	32,7	40,2	60,3	24,2	25,4
SPRTN$_{418-428}$	40,6	25	103,7	33,6	28,2
SART1$_{704-714}$	69,1	43,7	51,6	8,4	18
MKI67$_{2729-2739}$	32,3	47,5	48,8	7,8	17,5
IFI16$_{556-566}$	48	35,7	67,8	19,3	29,6
TDG$_{325-335}$	27,8	35,2	49,1	19,2	28,4
UBC$_{43-53}$	89,5	53,4	88,4	17,5	24,6
MKI67$_{1030-1040}$	75,7	47,2	75,2	14,9	20,7
PIAS1$_{310-320}$	63,5	38,2	68,1	19	19,6
SUMO2$_{2-12}$	30,3	31,5	42,9	8,7	18,8
KIF23$_{657-667}$	93,9	60,2	75,7	16,7	23,5
SP100$_{292-302}$	53,3	41,8	104,9	49,1	26,5
BAZ1B$_{821-831}$	62,1	40,2	94,3	25,5	34,8
PBRM1$_{1288-1298}$	60,6	44,1	84,8	15,5	28,4
ZNF106$_{1732-1742}$	50,6	36,5	98,6	55,4	30,3
CBX3$_{98-108}$	40	10,5	23,2	30,1	25,4
CBX4$_{275-285}$	73,1	44,9	79,6	24,9	21,5
MKI67$_{2004-2014}$	81,7	53,8	64,1	7,8	18,5
PARP1$_{462-472}$	83	51,4	83,3	19,6	20,8
TOP2B$_{1222-1232}$	68,1	47,7	74,1	40,7	32,5

	SENP1	SENP2	SENP5	SENP6	SENP7
UIMC1$_{15-25}$	71,5	44,6	56,1	2,7	12,5
XPC$_{76-86}$	61,4	40,4	62,4	20,9	14,8
ZNF451$_{827-837}$	78,7	55	92,4	51,3	29,8
PARP1$_{198-208}$	91,7	55	93,9	40,2	25,8
SUMO2$_{1-10}$	103,7	63,3	66	11,5	15,5
ZMYM2$_{83-103}$	95,5	64,9	78,2	19,5	23,8
SUMO1$_{32-42}$	35,9	30,6	59,7	20,9	12,1
UBC$_{58-68}$	69,7	46,5	84,2	12,7	23,3
PCNA$_{159-169}$	64,8	40,7	64,6	14,1	10,9
PML$_{375-385}$	83,3	49,4	67,1	11,3	12,5
NOP56$_{535-545}$	30,7	32,3	38,3	11	17
PIAS2$_{244-254}$	83,5	52,6	62,4	5,4	15,3
PIAS1$_{41-51}$	61	40	64,2	24,7	12,4
PIAS1$_{132-142}$	77,9	52,9	78,5	34,6	26,5
HNRNPM$_{383-393}$	48,4	34,9	92,1	18,9	23,7
SUMO1$_{12-22}$	99,3	56,9	64	2,9	14,7
TP53BP1$_{1429-1439}$	70,4	43,9	55,1	9,3	13,6
TP53BP1$_{1558-1568}$	57,4	40,7	62	22,2	21,2
RANBP2$_{2566-2576}$	68,8	32,6	59,3	23,5	21,2
CDK11B$_{238-248}$	56,9	41,6	55,9	24,9	28,6
MCM3$_{727-737}$	68,2	48,3	44,9	11,4	15

Concentration dependent FP assay

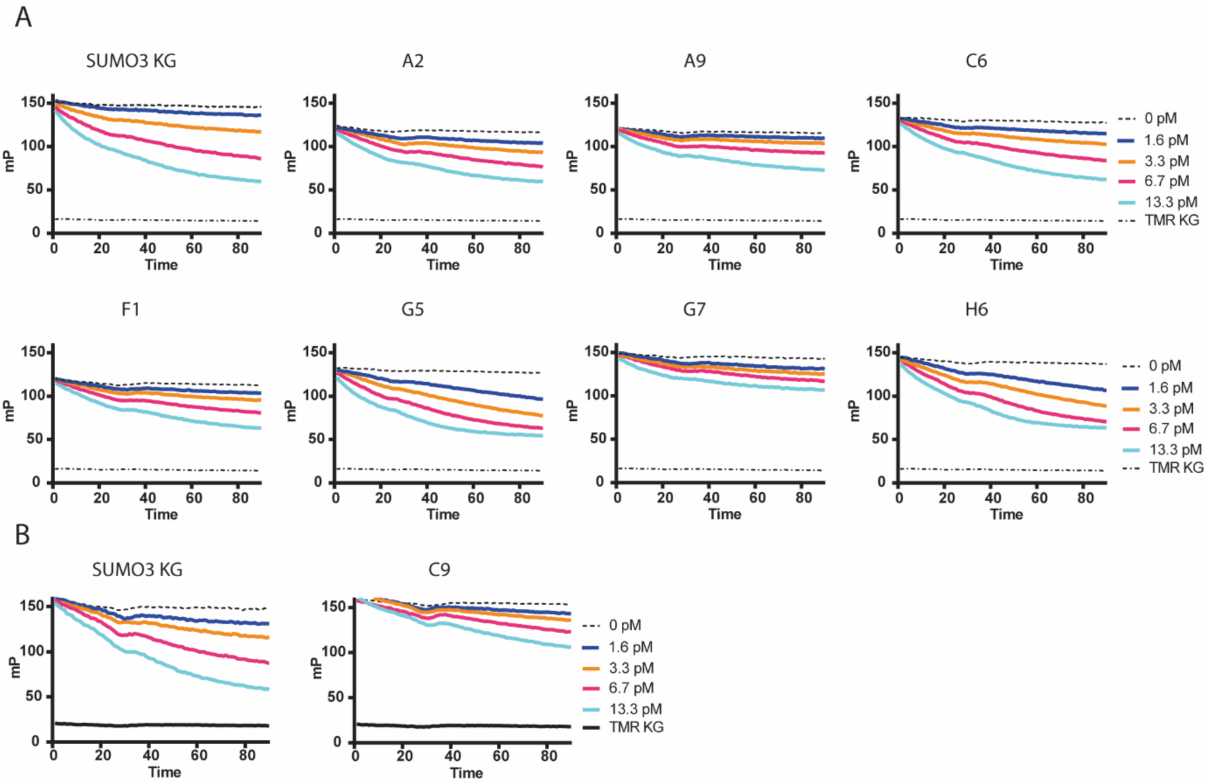

Figure S8. A. Time-course of probes **A2**, **A9**, **C6**, **F1**, **G5**, **G7**, **H6** and reference **SUMO TMR-KG** by increasing concentrations of SENP1. **B.** Time-course of probes **C9** and reference **SUMO TMR-KG** by increasing concentrations of SENP1.

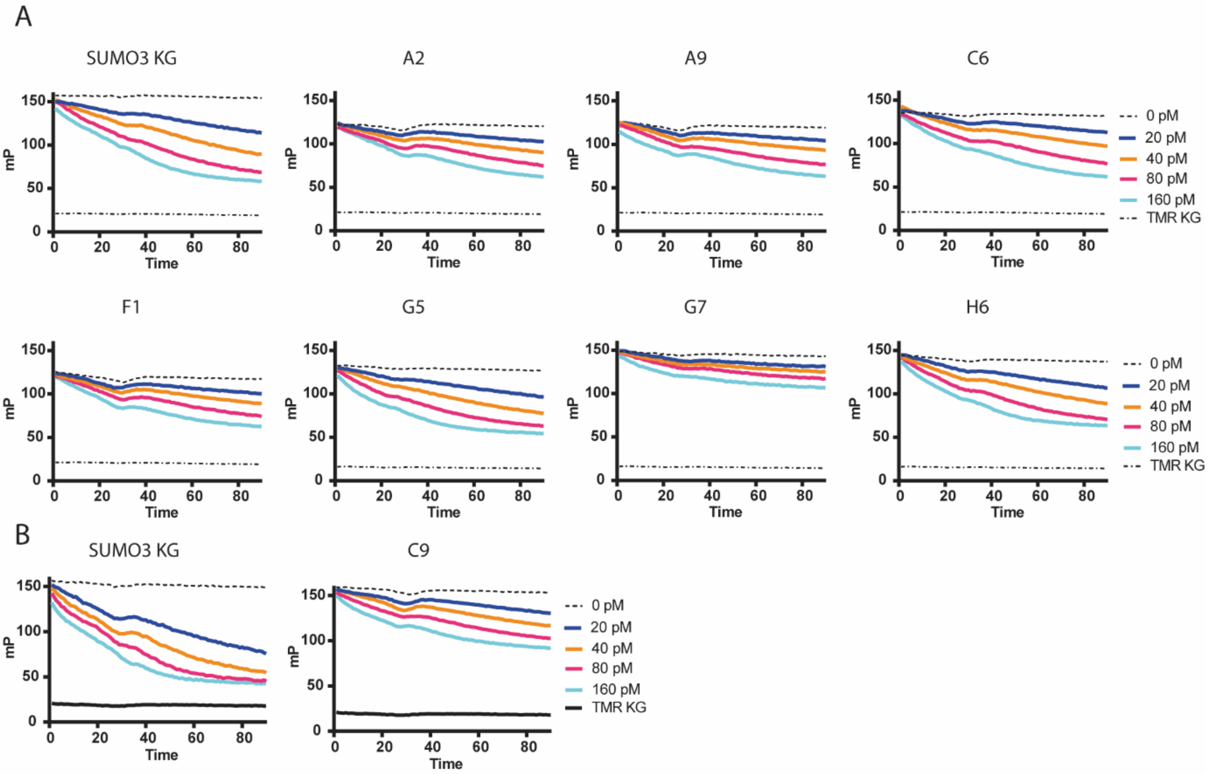

Figure S9. A. Time-course of probes **A2**, **A9**, **C6**, **F1**, **G5**, **G7**, **H6** and reference **SUMO TMR-KG** by increasing concentrations of SENP2. **B.** Time-course of probes **C9** and reference **SUMO TMR-KG** by increasing concentrations of SENP2.

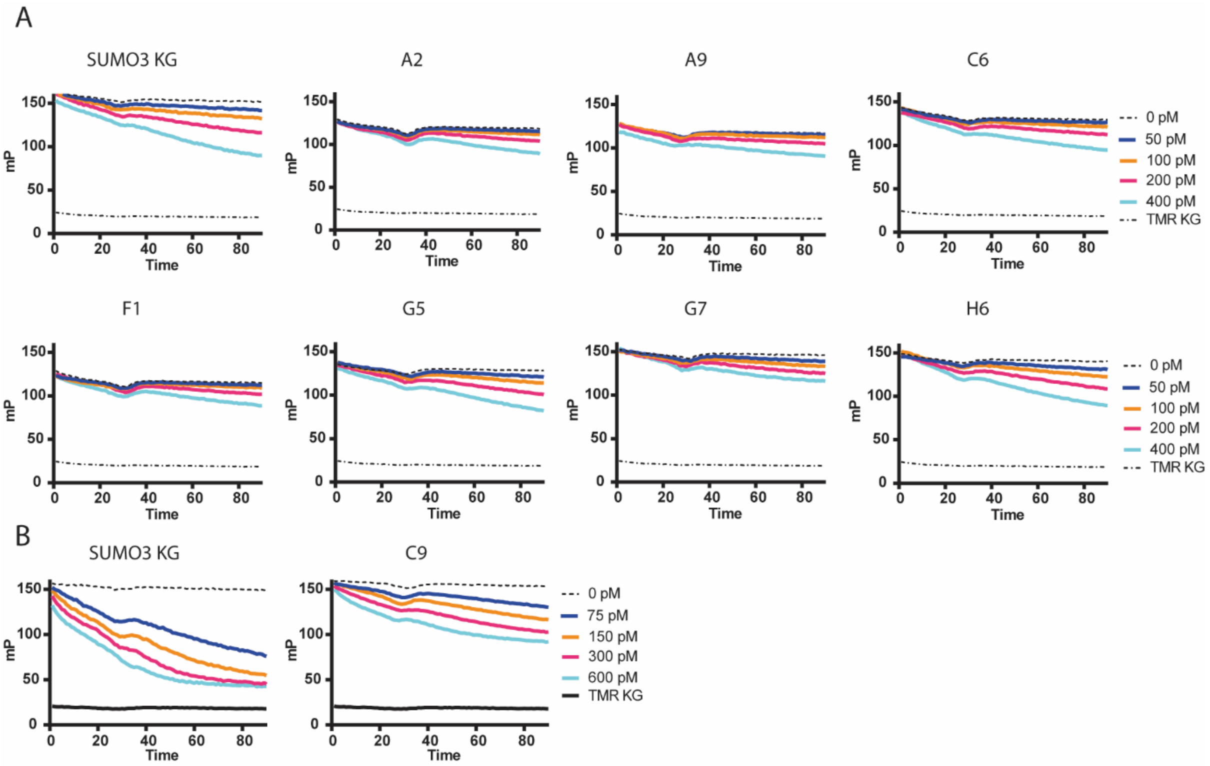

Figure S10. A. Time-course of probes **A2**, **A9**, **C6**, **F1**, **G5**, **G7**, **H6** and reference **SUMO TMR-KG** by increasing concentrations of SENP5. **B.** Time-course of probes **C9** and reference **SUMO TMR-KG** by increasing concentrations of SENP5.

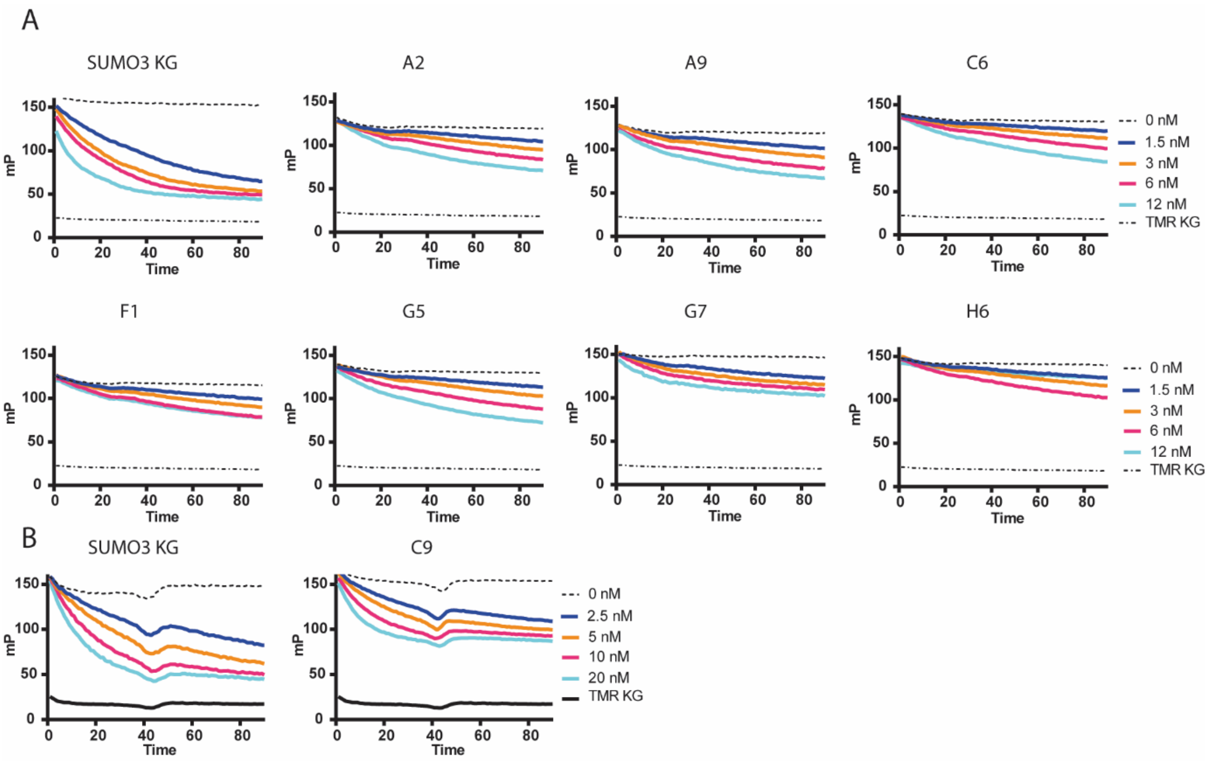

Figure S11. A. Time-course of probes **A2**, **A9**, **C6**, **F1**, **G5**, **G7**, **H6** and reference **SUMO TMR-KG** by increasing concentrations of SENP6. **B.** Time-course of probes **C9** and reference **SUMO TMR-KG** by increasing concentrations of SENP6.

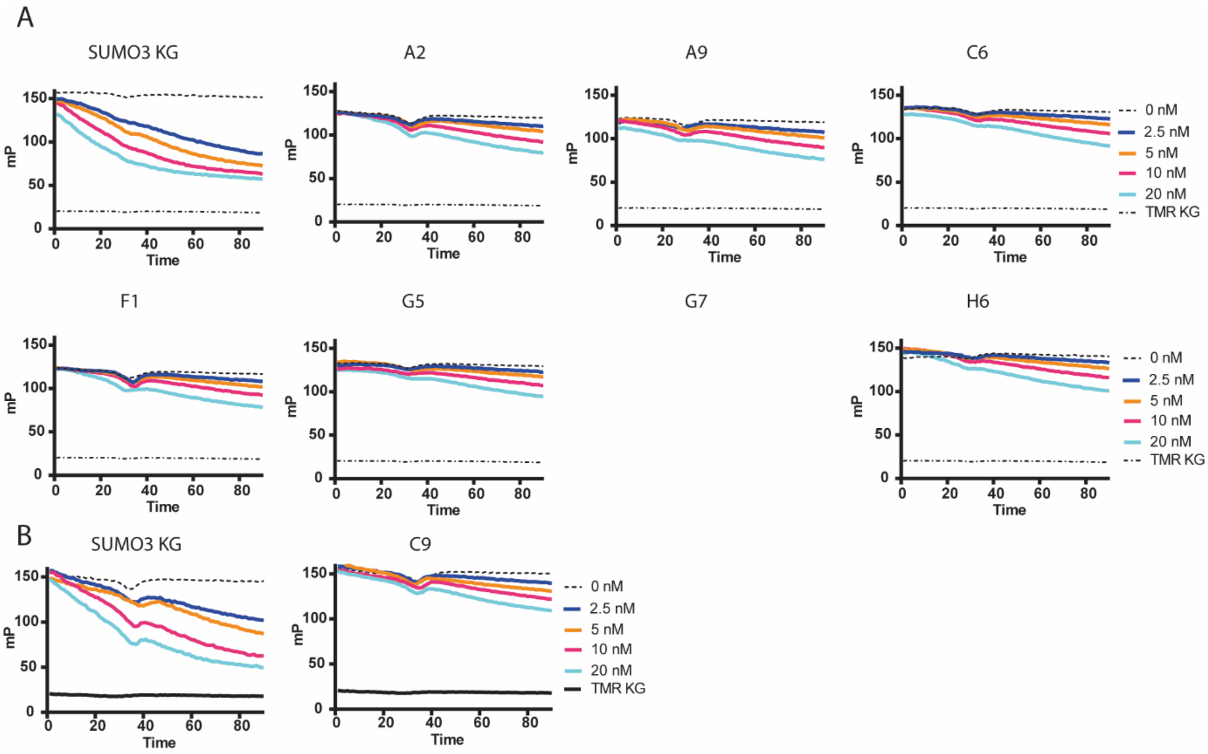

Figure S12. A. Time-course of probes **A2**, **A9**, **C6**, **F1**, **G5**, **G7**, **H6** and reference **SUMO TMR-KG** by increasing concentrations of SENP7. **B.** Time-course of probes **C9** and reference **SUMO TMR-KG** by increasing concentrations of SENP7.

Sequences:

SUMO3 sequence

```
1       MSEEKPKEGV KTENDHINLK VAGQDGSVVQ
31      FKIKRHTPLS KLMKAYCERQ GLSMRQIRFR
61      FDGQPINETD TPAQLEMEDE DTIDVFQQQT GG
```

SUMO2 sequence

```
1       MADEKPKEGV KTENNDHINL KVAGQDGSVV
31      QFKIKRHTPL SKLMKAYCER QGLSMRQIRF
61      RFDGQPINET DTPAQLEMED EDTIDVFQQQ TGG
```

SUMO1 sequence

```
1       MSDQEAKPST EDLGDKKEGE YIKLKVIGQD
31      SSEIHFKVKM TTHLKKLKES YCQRQGVPMN
61      SLRFLFEGQR IADNHTPKEL GMEEEDVIEV YQEQTGG
```

Table S6. Amino acids sequences of SUMO1,2 and 3

NAME	SEQUENCE
**SUMO3**	MSEEKPKEGVKTENDHINLKVAGQDGSVVQFKIKRHTPLSKLMKAYCERQGLSMRQIRFRFDGQPINETDTPAQLEMEDEDTIDVFQQQTGG
**SUMO1 K7**	XSDQEAZPSTEDLGDKKEGEYIKLKVIGQDSSEIHFKVKXTTHLKKLKESYSQRQGVPXNSLRFLFEGQRIADNHTPKELGXEEEDVIEVYQEQTGG
**SUMO1 K17**	XSDQEAKPSTEDLGDKZEGEYIKLKVIGQDSSEIHFKVKXTTHLKKLKESYSQRQGVPXNSLRFLFEGQRIADNHTPKELGXEEEDVIEVYQEQTGG
**SUMO1 K37**	XSDQEAKPSTEDLGDKKEGEYIKLKVIGQDSSEIHFZVKXTTHLKKLKESYSQRQGVPXNSLRFLFEGQRIADNHTPKELGXEEEDVIEVYQEQTGG
**SUMO2 K11**	XADEKPKEGVZTENNDHINLKVAGQDGSVVQFKIKRHTPLSKLXKAYSERQGLSXRQIRFRFDGQPINETDTPAQLEXEDEDTIDVFQQQTGG
**SUMO3 K11**	XSEEKPKEGVZTENDHINLKVAGQDGSVVQFKIKRHTPLSKLXKAYSERQGLSXRQIRFRFDGQPINETDTPAQLEXEDEDTIDVFQQQTGG
**SUMO3 K41**	XSEEKPKEGVKTENDHINLKVAGQDGSVVQFKIKRHTPLSZLXKAYSERQGLSXRQIRFRFDGQPINETDTPAQLEXEDEDTIDVFQQQTGG

X was incorporated as Fmoc-norleucine-OH and Z was incorporated as γ-thiolysine. Underlined dipeptide sequences were coupled as the respective pseudoproline dipeptides or DMB dipeptides.

Table S7. Amino acids sequences of SUMO1,2 and 3 with the corresponding dipeptides and special amino acids used in SPPS indicated.

Synthesis of SUMO1 derivates (SUMO1 thiolysine K7, K17 and K37)

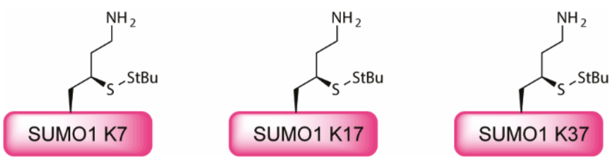

The synthesis was performed following general procedures (method 2) using 2-chlorotrityl resin (0.11 gram, 0.18 mmol/gram). The peptide was cleaved from the resin according to the general procedures and purified by RP-HPLC using a Phenomenex, Luna 100 Å, C8(2), 10 µm, 30 mm x 250 mm column (18-40 % B over 20 min, flow 30 mL/min) and lyophilized to afford the desired peptide as a white solid.
SUMO1 K7 thiolysine: 9.24 mg, 0.83 µmol, 4.2 % yield.
SUMO1 K17 thiolysine: 9.71 mg, 0.87 µmol, 4.4 % yield.
SUMO1 K37 thiolysine: 10.7 mg, 0.96 µmol, 4.8 % yield.

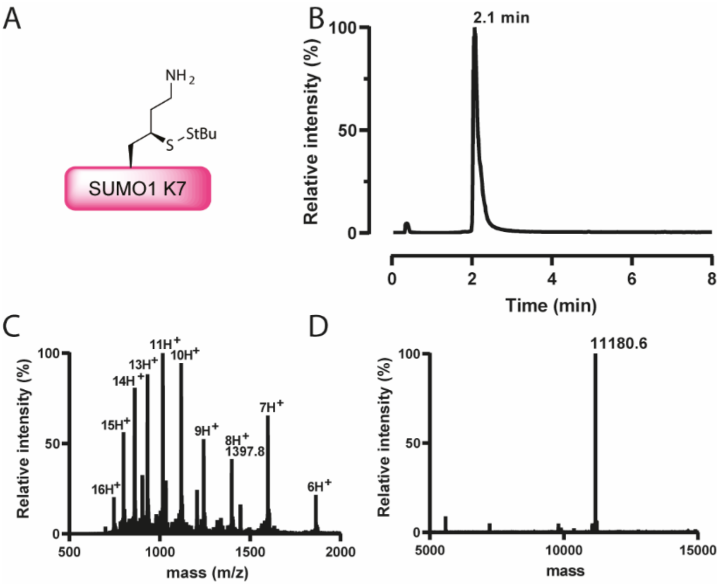

Figure S13. A. Schematic representation of SUMO1 K17 thiolysine. **B.** Total ion spectrum of SUMO1 K17 thiolysine, Rt 2.1 min. **C.** ESI spectrum of purified SUMO1 K17 thiolysine. Calculated Mass (average isotope composition): 11173.68; [M + 6H]$^{6+}$: 1863.28, [M + 7H]$^{7+}$: 1597.24, [M + 8H]$^{8+}$: 1397.71, [M + 9H]$^{9+}$: 1242.52, [M + 10H]$^{10+}$: 1118.37, [M + 11H]$^{11+}$: 1016.79, [M + 12H]$^{12+}$: 932.14, [M + 13H]$^{13+}$: 860.51. Observed: 11174.58; [M + 6H]$^{6+}$: 1863.43, [M + 7H]$^{7+}$: 1597.38, [M + 8H]$^{8+}$: 1397.84, [M + 9H]$^{9+}$: 1242.63, [M + 10H]$^{10+}$: 1118.47, [M + 11H]$^{11+}$: 1016.89, [M + 12H]$^{12+}$: 932.23, [M + 13H]$^{13+}$: 860.59. **D.** Deconvoluted mass of SUMO1 K17 thiolysine. Calculated: 11180.5, observed: 11180.6.

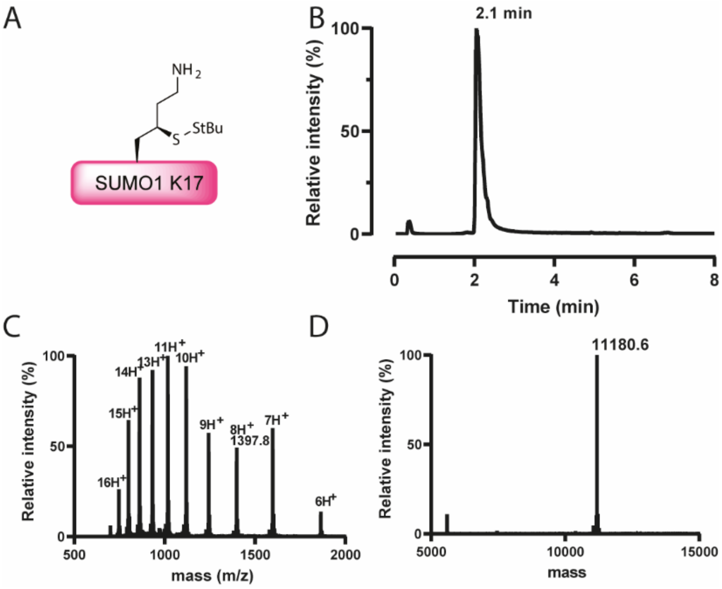

Figure S14. A. Schematic representation of SUMO1 K17 thiolysine. **B.** Total ion spectrum of SUMO1 K17 thiolysine, Rt 2.1 min. **C.** ESI spectrum of purified SUMO1 K17 thiolysine. Calculated Mass (average isotope composition): 11173.68; [M + 6H]$^{6+}$: 1863.28, [M + 7H]$^{7+}$: 1597.24, [M + 8H]$^{8+}$: 1397.71, [M + 9H]$^{9+}$: 1242.52, [M + 10H]$^{10+}$: 1118.37, [M + 11H]$^{11+}$: 1016.79, [M + 12H]$^{12+}$: 932.14, [M + 13H]$^{13+}$: 860.51. Observed: 11174.58; [M + 6H]$^{6+}$: 1863.43, [M + 7H]$^{7+}$: 1597.38, [M + 8H]$^{8+}$: 1397.84, [M + 9H]$^{9+}$: 1242.63, [M + 10H]$^{10+}$: 1118.47, [M + 11H]$^{11+}$: 1016.89, [M + 12H]$^{12+}$: 932.23, [M + 13H]$^{13+}$: 860.59. **D.** Deconvoluted mass of SUMO1 K17 thiolysine. Calculated: 11180.5, observed: 11180.6.

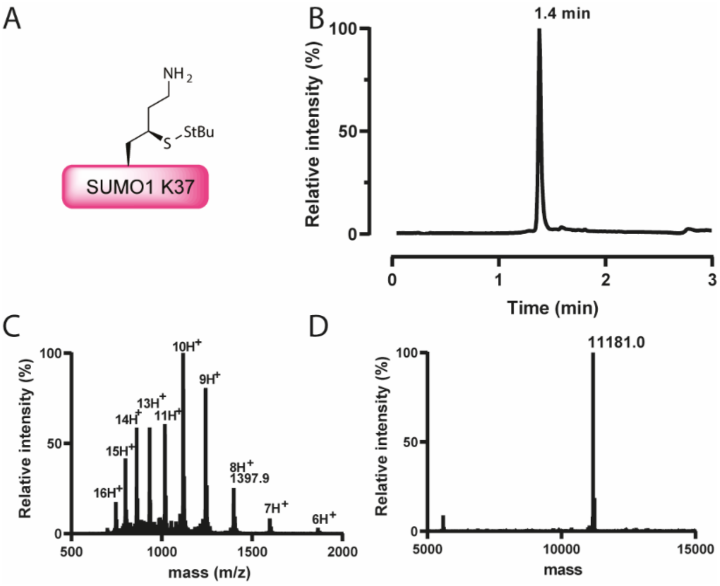

Figure S15. A. Schematic representation of SUMO1 K37 thiolysine. **B.** Total ion spectrum of SUMO1 K37 thiolysine, Rt 1.4 min. **C.** ESI spectrum of purified SUMO1 K37 thiolysine. Calculated Mass (average isotope composition): 11173.68; $[M + 6H]^{6+}$: 1863.28, $[M + 7H]^{7+}$: 1597.24, $[M + 8H]^{8+}$: 1397.71, $[M + 9H]^{9+}$: 1242.52, $[M + 10H]^{10+}$: 1118.37, $[M + 11H]^{11+}$: 1016.79, $[M + 12H]^{12+}$: 932.14, $[M + 13H]^{13+}$: 860.51. Observed: 11175.3; $[M + 6H]^{6+}$: 1863.55, $[M + 7H]^{7+}$: 1597.46, $[M + 8H]^{8+}$: 1397.91, $[M + 9H]^{9+}$: 1242.71, $[M + 10H]^{10+}$: 1118.43, $[M + 11H]^{11+}$: 1016.94, $[M + 12H]^{12+}$: 932.29, $[M + 13H]^{13+}$: 860.64. **D.** Deconvoluted mass of SUMO1 K37 thiolysine. Calculated: 11180.5, observed: 11181.0.

Synthesis of SUMO2 thiolysine K11

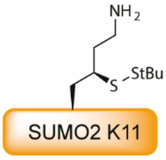

The synthesis was performed following general procedures (method 2) using 2-chlorotriyl resin (0.11 gram, 0.18 mmol/gram). The protected polypeptide was cleaved from the resin by treatment with 3 x 5 mL of DCM/TFE 3:1 v/v for 1 hour and followed by filtration. The combined filtrates were concentrated *in vacuo* and co-evaporated with DCE 3x and dried under high vacuum. The protected peptide (1 equiv.) was dissolved in DCM (10 mL) and methyl 3-mercaptopropionate (8.8 µL, 80 µmol, 4 equiv.) was coupled using PyAOP (42 mg, 80 µmol, 4 equiv.) and DIPEA (27.8 µL, 160 µmol, 8 equiv.) for 16 hours. Thereafter, the solvents were removed *in vacuo* and the protecting groups was cleaved according to the general procedures. The crude peptide was purified by RP-HPLC using Phenomenex, Luna 100 Å, C8(2), 10 µm, 30 mm x 250 mm column (18-40 % B over 20 min, flow 30 mL/min) and lyophilized to afford the desired peptide (16.28 mg, 7.65% yield).

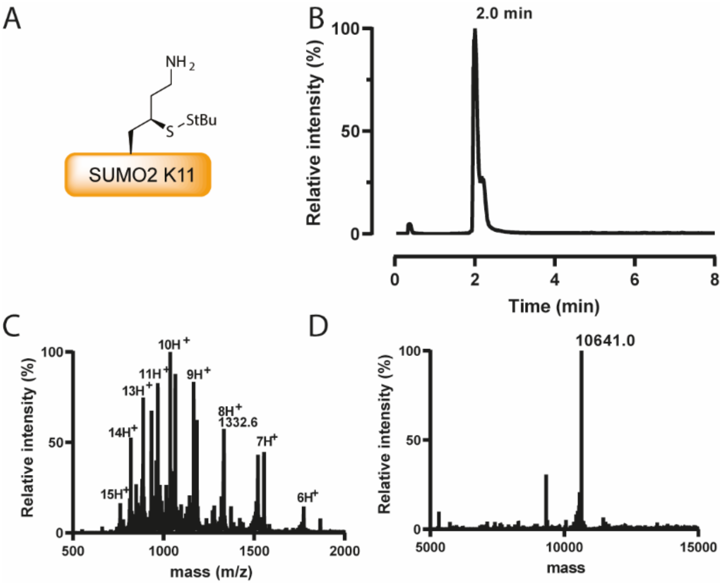

Figure S16. A. Schematic representation of SUMO2 K11 thiolysine. **B.** Total ion spectrum of SUMO2 K11 thiolysine, Rt 2.0 min. **C.** ESI spectrum of purified SUMO2 K11 thiolysine. Calculated Mass (average isotope composition): 10634.44; $[M + 6H]^{6+}$: 1773.41, $[M + 7H]^{7+}$: 1520.20, $[M + 8H]^{8+}$: 1330.31, $[M + 9H]^{9+}$: 1182.60, $[M + 10H]^{10+}$: 1064.44, $[M + 11H]^{11+}$: 967.76, $[M + 12H]^{12+}$: 887.20, $[M + 13H]^{13+}$: 819.04. Observed: 10633.44; $[M + 6H]^{6+}$: 1773.24, $[M + 7H]^{7+}$: 1520.06, $[M + 8H]^{8+}$: 1332.55, $[M + 9H]^{9+}$: 1182.36, $[M + 10H]^{10+}$: 1064.35, $[M + 11H]^{11+}$: 967.68, $[M + 12H]^{12+}$: 887.04, $[M + 13H]^{13+}$: 819.12. **D.** Deconvoluted mass of SUMO2 K11 thiolysine. Calculated: 10640.8, observed: 10641.0.

Synthesis of SUMO3 thiolysine derivatives (K11, K41)

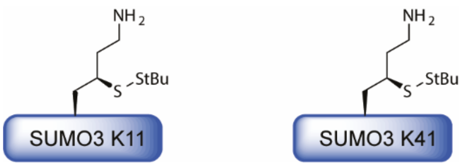

The synthesis was performed following general procedures (method 2) using 2-chlorotriyl resin (0.11 gram, 0.18 mmol/gram). The peptide was cleaved from the resin according to the general procedures and purified by RP-HPLC using a Phenomenex, Luna 100 Å, C8(2), 10 µm, 30 mm x 250 mm column (18-40 % B over 20 min, flow 30 mL/min) and lyophilized to afford the desired peptide as a white solid.

SUMO3 K11 thiolysine: 14.74 mg , 0.13 µmol, 7.0 % yield
SUMO3 K41 thiolysine: 16.53 mg, 0.15 µmol, 7.8 % yield

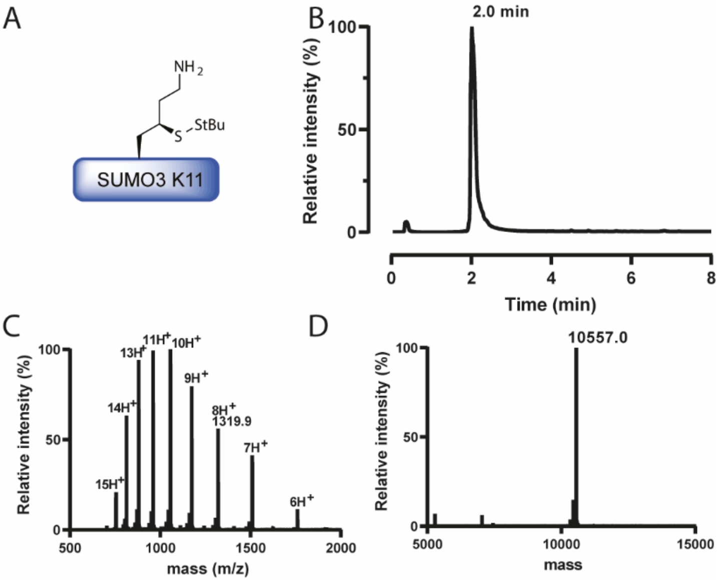

Figure S17. A. Schematic representation of SUMO3 K11 thiolysine. **B.** Total ion spectrum of SUMO3 K11 thiolysine, Rt 2.0 min. **C.** ESI spectrum of purified SUMO3 K11 thiolysine. Calculated Mass (average isotope composition): 10550.40; $[M + 6H]^{6+}$: 1759.4, $[M + 7H]^{7+}$: 1508.2, $[M + 8H]^{8+}$: 1319.8, $[M + 9H]^{9+}$: 1173.27, $[M + 10H]^{10+}$: 1056.04, $[M + 11H]^{11+}$: 960.13, $[M + 12H]^{12+}$: 880.20, $[M + 13H]^{13+}$: 812.57. Observed: 10551.42; $[M + 6H]^{6+}$: 1759.57, $[M + 7H]^{7+}$: 1508.34, $[M + 8H]^{8+}$: 1319.93, $[M + 9H]^{9+}$: 1173.38, $[M + 10H]^{10+}$: 1056.14, $[M + 11H]^{11+}$: 960.13, $[M + 12H]^{12+}$: 880.29, $[M + 13H]^{13+}$: 812.66. **D.** Deconvoluted mass of SUMO2 K11 thiolysine. Calculated: 10556.7, observed: 10557.0.

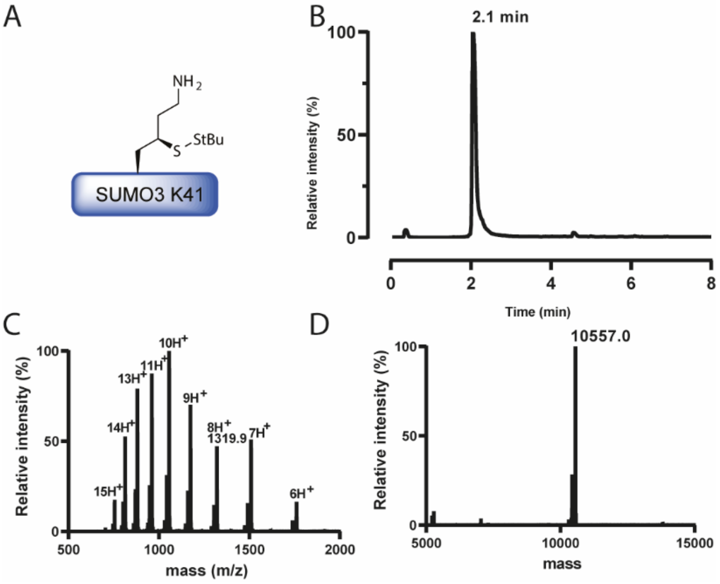

Figure S18. A. Schematic representation of SUMO3 K41 thiolysine. **B.** Total ion spectrum of SUMO3 K41 thiolysine, Rt 2.1 min. **C.** ESI spectrum of purified SUMO3 K41 thiolysine. Calculated Mass (average isotope composition): 10550.40; $[M + 6H]^{6+}$: 1759.4, $[M + 7H]^{7+}$: 1508.2, $[M + 8H]^{8+}$: 1319.8, $[M + 9H]^{9+}$: 1173.27, $[M + 10H]^{10+}$: 1056.04, $[M + 11H]^{11+}$: 960.13, $[M + 12H]^{12+}$: 880.20, $[M + 13H]^{13+}$: 812.57. Observed: 10551.36; $[M + 6H]^{6+}$: 1759.56, $[M + 7H]^{7+}$: 1508.34, $[M + 8H]^{8+}$: 1319.93, $[M + 9H]^{9+}$: 1173.38, $[M + 10H]^{10+}$: 1056.14, $[M + 11H]^{11+}$: 960.22, $[M + 12H]^{12+}$: 880.29, $[M + 13H]^{13+}$: 812.65. **D.** Deconvoluted mass of SUMO2 K11 thiolysine. Calculated: 10557.0, observed: 10557.0.

SUMO dimer synthesis

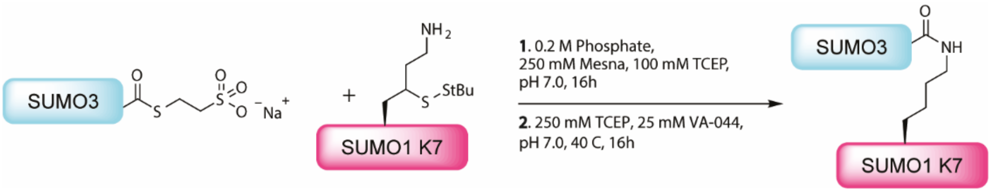

SUMO dimers were synthesized using the same protocol as was established for the 96-well synthesis with some minor adjustments. In short, SUMO1 was dissolved in 20 mM DMSO followed by dilution into buffer containing 0.2 M Na_2HPO_4, 250 mM MESNa and 0.15 M NaCl, 50 mM TCEP pH 7.95 for a final concentration of 1 mM. Peptide **1** (1.5 equiv.) was added as solid to the reaction mixture followed by pH adjustment to pH 7.6. The reaction mixture was shaken at 37 °C for 16 hours followed by gel analysis of the reaction. Followed by the addition of an equal amount of 0.2 M Na_2HPO_4, 500 mM TCEP and 50 mM VA-044 in MilliQ, resulting in a final buffer concentration of 0.2 M Na_2HPO_4, 250 mM TCEP, 125 mM MESNa and 25 mM VA-044 in MilliQ. The reaction was shaken at 350 rpm at 40 °C for 16 hours. Thereafter, the reaction mixture was diluted with 6 M Gdn HCl and purified by RP-HPLC using a Phenomenex, Jupiter 300 Å, C4, 5 µm, 10 mm x 250 mm column. The fractions containing the product were pooled and lyophilized before final purification using SEC. The peptides were dissolved in 2 M Gdn HCl and purified on a Superdex 75 10/300 GL column (GE Healthcare). Fractions were analyzed by SDS-page and pure fractions were pooled, followed by concentration determination by Nanodrop. Yields are not determined.

SUMO1 K7: 3.91 mg, SUMO3 (**1**): 6.25 mg.
SUMO1 K17: 3.22 mg, SUMO3 (**1**): 5.85 mg.
SUMO1 K37: 3.83 mg, SUMO3 (**1**): 6.48 mg.

SUMO2 K11: 4.32 mg, SUMO3 (**1**): 7.09 mg.

SUMO3 K41: 5.53 mg, SUMO3 (**1**): 6.29 mg.
SUMO3 K11: 3.74 mg, SUMO3 (**1**): 4.63 mg.

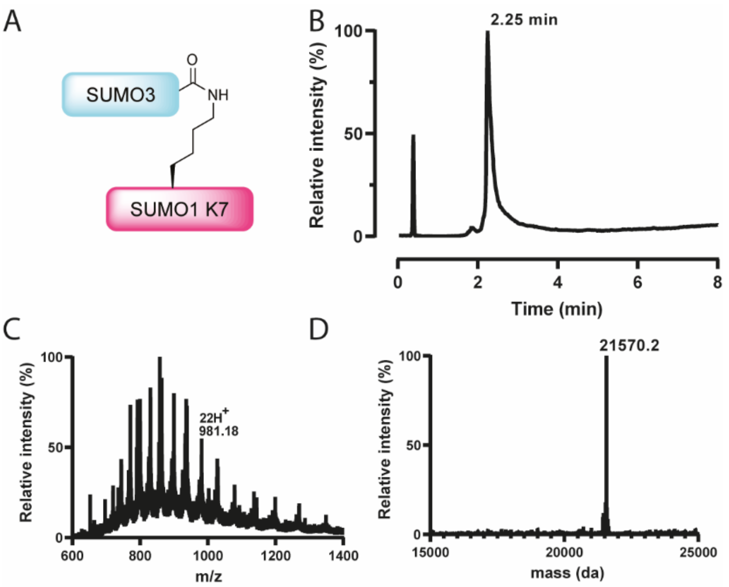

Figure S19. A. Schematic representation of S3-S1 K7 dimer. **B.** Total ion spectrum (LC-MS method C4) of S3-S1 K7, Rt 2.25 min. **C.** ESI spectrum of purified S3-S1 K7. **D.** Deconvoluted mass of S3S1 K7. Calculated: 21567.0, observed: 21570.2 .

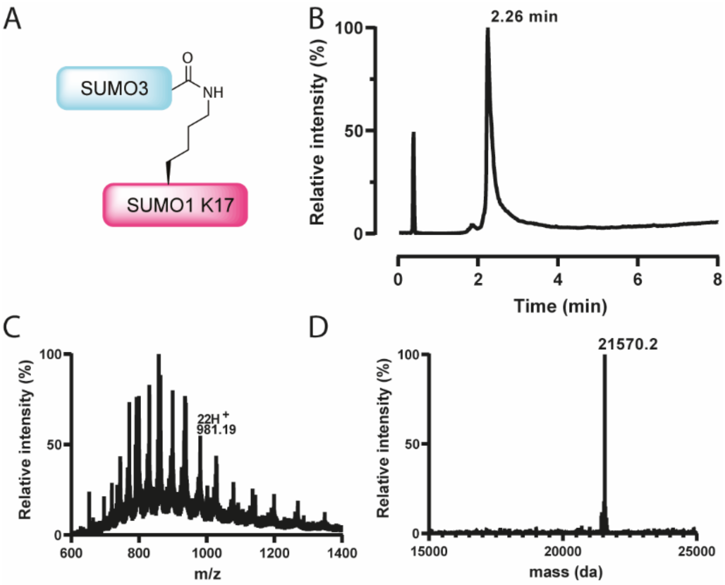

Figure S20. A. Schematic representation of S3-S1 K17 dimer. **B.** Total ion spectrum (LC-MS method C4) of S3-S1 K17, Rt 2.26 min. **C.** ESI spectrum of purified S3-S1 K17. **D.** Deconvoluted mass of S3-S1 K7. Calculated: 21567.0, observed: 21570.2.

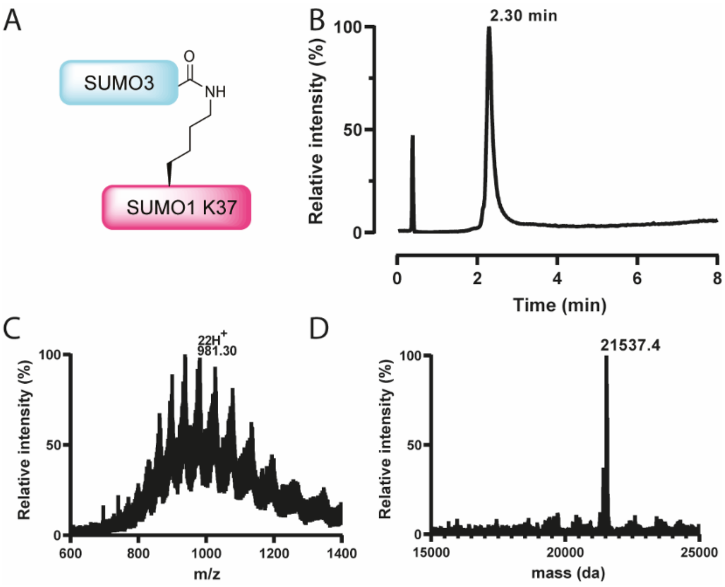

Figure S21. A. Schematic representation of S3-S1 K37 dimer. **B.** Total ion spectrum (LC-MS method C4) of S3-S1 K37, Rt 2.30 min. **C.** ESI spectrum of purified S3-S1 K37. **D.** Deconvoluted mass of S3-S1 K37. Calculated: 21567.0, observed: 21537.4. The mass observed is from S3-S1 K37 where the native cysteine is desulfurized.

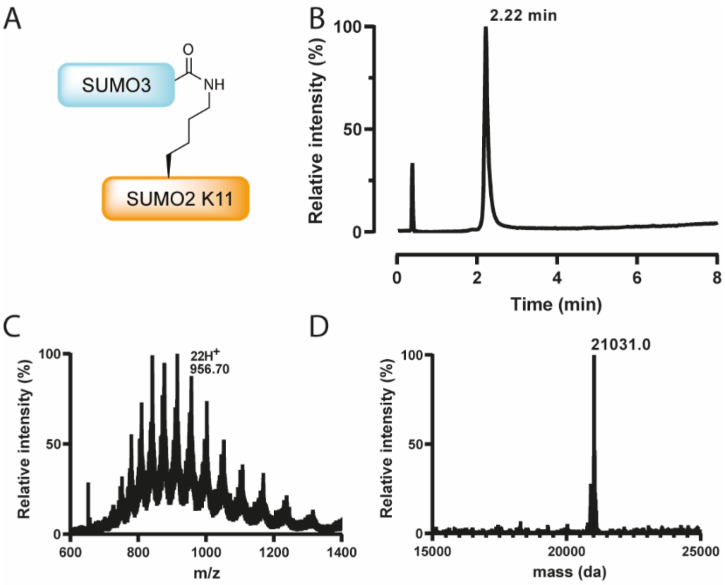

Figure S22. A. Schematic representation of S3-S2 K11 dimer. **B.** Total ion spectrum (LC-MS method C4) of S3-S2 K11, Rt 2.22 min. **C.** ESI spectrum of purified S3-S2 K11. **D.** Deconvoluted mass of S3-S2 K11. Calculated: 21027.3, observed: 21031.0.

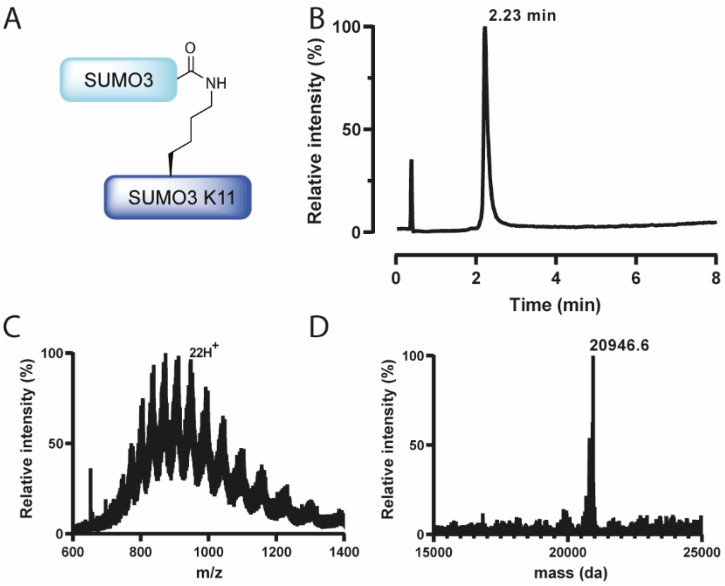

Figure S23. A. Schematic representation of S3-S3 K11 dimer. **B.** Total ion spectrum (LC-MS method C4) of S3-S3 K11, Rt 2.23 min. **C.** ESI spectrum of purified S3-S3 K11. **D.** Deconvoluted mass of S3-S3 K11. Calculated: 20943.2, observed: 20946.6.

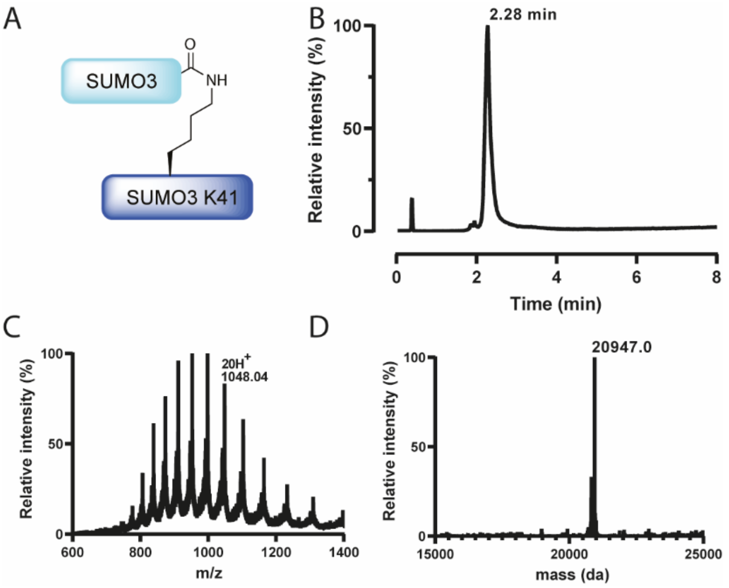

Figure S24. A. Schematic representation of S3-S3 K41 dimer. **B.** Total ion spectrum (LC-MS method C4) of S3-S3 K41, Rt 2.28 min. **C.** ESI spectrum of purified S3-S3 K41. **D.** Deconvoluted mass of S3-S3 K41. Calculated: 20943.2, observed: 20947.0.

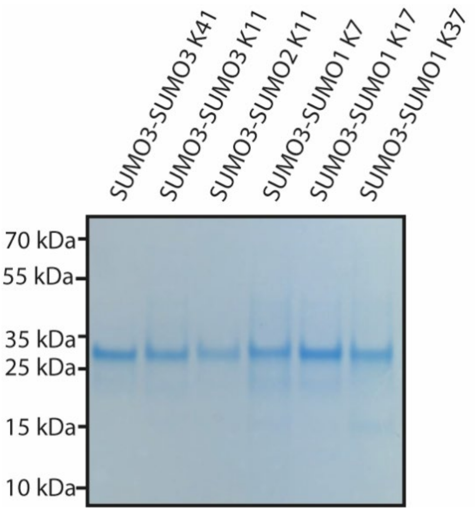

Figure S25. SDS-page analysis of all SUMO3 dimers.

Fluorescence Polarization SENP Assay

Fluorescent polarization (FP) assays were performed as described by Geurink et al. Assays were performed in TRIS buffer (50 mM Tris.HCl, pH 7.5, 5 mM DTT, 100 mM NaCl, 1 mg/mL 3-{Dimethyl[3-(3α,7α,12α-trihydroxy-5β-cholan-24-amido)propyl]azaniumyl}propane-1-sulfonate (CHAPS) and 0.5 mg/mL bovine gamma globulin (BGG)). All probes (150 nL of 40 µM in DMSO, final concentration 400 nM) were dispensed into ‘‘non-binding surface flat bottom low flange’’ black 384-well plates (Corning) plates using an ECHO 550 Liquid Handler (Labcyte Inc.) acoustic dispenser. Buffer was predispensed (10 µL /well) and the reaction was started by the addition of enzyme (SENP1, SENP2, SENP5, SENP6, and SENP7) (5 µL /well, final concentration in table S6). The plate was centrifuged (1 min at 1,500 rpm) prior to the measurement. FP of the TAMRA fluorophore was measured every 81 seconds for 90 minutes on a Pherastar plate reader (BMG LABTECH GmbH, Germany) with 540-590-590 FP module (λex = 540 nm with detection of polarization at λem = 590 nm). The obtained data was analyzed by GraphPad prism (version 9.0.1).

In vitro SENP assay

Qualitative in vitro linkage specificity assays were performed as described by Mevissen et al. and Licchesi et al. In short, to diSUMO probes dissolved in 100 mM NaCl, 100 mM Tris (pH 7.4), and 2 mM TCEP, SENP was added according to the concentrations in table S7. Pilot experiments identified the required SENP concentration that resulted in cleavage of the preferred chain type. 2 µg diSUMO was taken out of the reaction mixture at the indicated time points. The reaction is resolved on 4-12% SDS-page gradient gels (Invitrogen) run in MES buffer (Invitrogen) and stained by Instant Blue (Abcam) protein stain.
